# Cutting-Edge iPSC-Based Approaches in Studying Host—Microbe Interactions in Neuropsychiatric Disorders

**DOI:** 10.3390/ijms251810156

**Published:** 2024-09-21

**Authors:** Marija Mihailovich, Svetlana Soković Bajić, Miroslav Dinić, Jelena Đokić, Milica Živković, Dušan Radojević, Nataša Golić

**Affiliations:** 1 Institute of Molecular Genetics and Genetic Engineering (IMGGE), University of Belgrade, Vojvode Stepe 444a, 11042 Belgrade, Serbia; svetlana.sokovic@imgge.bg.ac.rs (S.S.B.); mdinic@imgge.bg.ac.rs (M.D.); jelena.djokic@imgge.bg.ac.rs (J.Đ.); milicanikolic@imgge.bg.ac.rs (M.Ž.); dradojevic@imgge.bg.ac.rs (D.R.); 2 Human Technopole, Palazzo Italia, Viale Rita Levi-Montalcini, 1, 20157 Milan, Italy

**Keywords:** microbiota, neuroinflammation, gut–brain axis, probiotics, targeted culturomics, dysbiosis, iPSC-based gut barrier, blood–brain barrier and gut–brain axis models

## Abstract

Gut microbiota (GM), together with its metabolites (such as SCFA, tryptophan, dopamine, GABA, etc.), plays an important role in the functioning of the central nervous system. Various neurological and psychiatric disorders are associated with changes in the composition of GM and their metabolites, which puts them in the foreground as a potential adjuvant therapy. However, the molecular mechanisms behind this relationship are not clear enough. Therefore, before considering beneficial microbes and/or their metabolites as potential therapeutics for brain disorders, the mechanisms underlying microbiota–host interactions must be identified and characterized in detail. In this review, we summarize the current knowledge of GM alterations observed in prevalent neurological and psychiatric disorders, multiple sclerosis, major depressive disorder, Alzheimer’s disease, and autism spectrum disorders, together with experimental evidence of their potential to improve patients’ quality of life. We further discuss the main obstacles in the study of GM–host interactions and describe the state-of-the-art solution and trends in this field, namely “culturomics” which enables the culture and identification of novel bacteria that inhabit the human gut, and models of the gut and blood–brain barrier as well as the gut–brain axis based on induced pluripotent stem cells (iPSCs) and iPSC derivatives, thus pursuing a personalized medicine agenda for neuropsychiatric disorders.

## 1. Introduction

Gut microbiota (GM) is composed of 10^14^ microorganisms, including bacteria, archaea, eukaryotes, and viruses. High-throughput sequencing methods have revolutionized the field of GM. Amplicon metagenomic sequencing (16S rDNA) allowed for the identification of bacteria, archaea, and fungi, while shotgun metagenomic sequencing enabled the generation of the complete genomes of all present microorganisms [1]. MetaHit and the Human Microbiome Project have provided a comprehensive overview of human GM [2,3], which resulted in an integrated gene catalogue composed of 9,879,896 human GM genes [3].

GM colonization occurs early in life; it begins in the womb and is followed by rapid colonization immediately after birth [4]. The final composition of an individual’s GM depends on environmental factors such as diet, stress, and lifestyle [5]. Diseases, major changes in diet, and especially the use of antibiotics dramatically alter the composition and diversity of GM, leading to dysbiosis, which is closely associated with various diseases [6]. Although there is still no consensus regarding what constitutes “normal microbiota” after decades of studying human GM, it is generally accepted that GM diversity is a good indicator of a “healthy gut” [7].

GM is an ecosystem that co-evolved with the host. Intestinal microbiota have very dynamic relationships, both with each other and with the host. A growing body of evidence reveals the importance of GM for human health. Gut bacteria produce various metabolites that integrate with host metabolites, participating in the breakdown of indigestible carbohydrates and in the development and functioning of the host’s immune and nervous systems [8,9]. The most important are discussed below.

### 1.1. Short-Chain Fatty Acids (SCFAs)

SCFAs (acetate, propionate, and butyrate) are produced by anaerobic GM through the fermentation of dietary fibers. SCFAs perform several important functions in the gut lumen, such as maintaining redox balance, preserving gut barrier integrity, and promoting the production of gut hormones [10]. They are among the most well-described microbial metabolites with anti-inflammatory and immunoregulatory potential. Mechanistically, SCFAs act via histone deacetylase (HDAC) inhibition and/or G-coupled protein receptors (GPR41, GPR43, and GPR109A). Butyrate and propionate attenuate LPS-induced immune responses through HDAC inhibition and regulate innate immune cell migration and functions [11,12]. They also stimulate the proliferation and function of Tregs, which are crucial for maintaining peripheral tolerance, preventing autoimmune diseases, and limiting chronic inflammatory conditions [13,14]. Additionally, by inhibiting HDAC activity, SCFAs promote the maturation of microglial cells and influence neuronal functioning [15]. Several important microbiota taxa, such as *Akkermansia*, *Bifidobacteria*, *Faecalibacterium*, *Lachnospiraceae*, *Lactobacillus*, and *Ruminococcus*, produce SCFAs [16].

### 1.2. Tryptophan

Tryptophan is an essential amino acid necessary for protein synthesis, growth, and health in animals and humans. It is the sole precursor of serotonin (5-hydroxytryptamine, 5-HT), a key monoamine neurotransmitter that modulates central neurotransmission and enteric physiological functions, such as emotional control, food intake, sleep, and pain processing [17]. However, only 10% of serotonin resides in the central nervous system (CNS), while 90% is located in the gastrointestinal (GI) tract. Under physiological conditions, serotonin cannot cross the blood–brain barrier (BBB) [17]. More than 90% of tryptophan is oxidized into kynurenine via the kynurenine pathway, which regulates tryptophan availability by clearing its excess. Kynurenine can be further metabolized through the kynurenic acid (KYNA) and quinolinic acid (QUIN) pathways [18]. Both KYNA and QUIN can cross the BBB and are involved in various physiological and pathological processes in both the brain and GI tract. KYNA acts as an N-methyl D-aspartate (NMDA) receptor antagonist, while QUIN functions as an NMDA receptor agonist, making KYNA a neuroprotector and QUIN a neurotoxin. Kynurenines are also inflammatory mediators. Alongside the kynurenine pathway, tryptophan can be metabolized by indoleamine-2,3-dioxygenase (IDO) into indole, a process activated by immune stimuli (Gao, Mu et al. 2020) [17]. Dietary tryptophan undergoes complex metabolic transformations regulated by GM [19]. GM can modulate the availability of tryptophan through tryptophan decarboxylation, generation of indole and its derivatives, and serotonin production [19]. Increased LPS-dependent inflammation, related to a reduction in Firmicutes and subsequent decrease in SCFAs, is shown to promote IDO activity and the generation of kynurenine from tryptophan [19].

### 1.3. Dopamine

Dopamine, the most abundant catecholamine neurotransmitter in the brain, is synthesized from dietary tyrosine and transported to the brain via the BBB. In the gut, certain bacteria, such as *Staphylococcus*, can convert L-DOPA to dopamine, with over 50% of the body’s dopamine being synthesized in the gut. This neurotransmitter plays a crucial role in regulating various physiological functions, including mucosal blood flow, motility, and gastric secretion.

### 1.4. γ-Amino Butyric Acid (GABA)

GABA is the main inhibitory neurotransmitter and participates in multiple physiological processes in the CNS by reducing neuronal excitability. It is linked to mood disorders, anxiety, and depression [20]. Many lactic acid bacteria (LAB) convert glutamate from food into GABA via the glutamate (glutamic acid) decarboxylase (GAD) enzyme, which is activated at low pH to increase tolerance to acidic conditions in the gut [21]. *Lactobacillus brevis* and *Bifidobacterium dentium* are efficient GABA producers [22,23]. GABA produced by the microbiota can cross the gut barrier and bind to receptors widely distributed on enteric neurons and the vagus nerve, thereby directly impacting the nervous system [20]. GABA also regulates GI activity [24].

### 1.5. Gut Microbiota–Brain Cross-Talk

**Figure 1:**
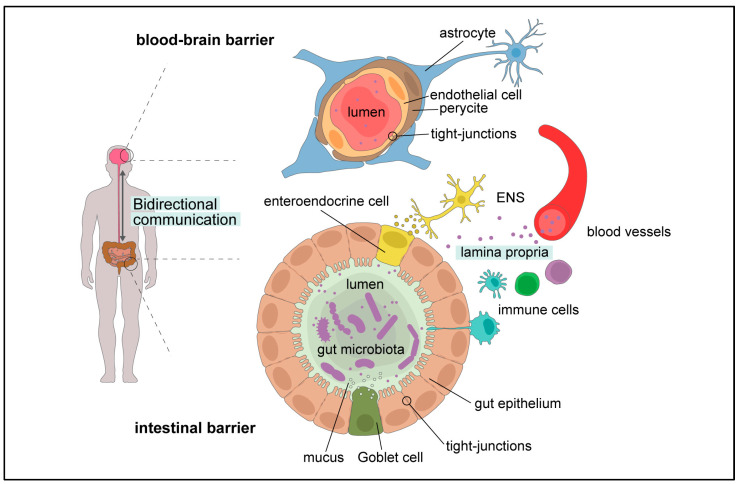
Bidirectional communication network between the gut and brain depicting cross-talk between two semi-permeable barriers: the intestinal barrier and blood–brain barrier; ENS—enteric nervous system.

The gut–brain axis (GBA) is a bidirectional communication network between the gut and brain, involving neuronal, endocrine, metabolic, and immune signaling molecules that must pass through two semi-permeable barriers: the gut barrier and the BBB [25], the existence of which is widely accepted today (Figure 1). One of the big challenges in the field is understanding the molecular mechanisms underlying the cross-talk between the GM and the host, and deciphering the impact of microbial-derived metabolites on the GBA. GM metabolites affect the brain directly (direct stimulation of receptors and activation of downstream signaling pathways and epigenetic regulation of histone acetylation) and indirectly (stimulation of neural, endocrine, and immune modulators).

Inflammation (chronic and/or neuroinflammation) is associated with many, if not all, neurological and psychiatric disorders. The first line of defense against many common microorganisms is provided by the innate immune response, mediated by macrophages and neutrophils. This response limits infection spread but does not provide the memory of the action. In addition to the innate immune response, there is also an adaptive immune response that is specific to microbial antigens (peptides expressed by specific microorganisms). Microbial-associated molecular patterns, such as lipopolysaccharides (LPSs), which are components of the outer membrane of Gram-negative bacteria, trigger the host immune response by stimulating antigen-presenting cells (e.g., dendritic cells). These cells then present antigen to antigen-specific lymphocytes, initiating the adaptive immune response. Additionally, some microbial metabolites induce immunosuppressive cells, such as regulatory T and B lymphocytes (Treg and Breg cells). Some activated lymphocytes differentiate into memory cells, ensuring a faster and more effective immune response or tolerance to the same antigens. These mechanisms represent fundamental aspects of microbiota–host cohabitation. Although specificity is a crucial feature of the adaptive immune response, individuals with specific genetic predispositions, when exposed to certain environmental factors, may inadequately direct, stimulate, or attenuate specific immune system cells, leading to chronic inflammation and the development or acceleration of associated neuropsychiatric disorders. Changes in microbiota and their metabolites have emerged as critical environmental factors that could trigger dysregulation of the immune response.

Bacterial strains that produce metabolites or neurotransmitters, such as SCFAs, GABA, serotonin, dopamine, etc., which influence CNS function, are referred to as psychobiotics. These neuromodulators affect sleep, appetite, mood, and cognition [26]. Although the precise mechanisms by which GM affects the CNS remain elusive, it is clear that GM dysbiosis is strongly associated with the development of neurological and psychiatric disorders, such as multiple sclerosis, major depressive disorder, Alzheimer’s disease, and autism spectrum disorder, which are primarily emphasized in this review. We will also discuss the main hurdles in studying GM–host interactions: (i) the problem of the cultivation of psychobiotics, since many human gut bacteria associated with brain disorders are strict anaerobes (e.g., SCFA producers) or have unknown cultivation conditions, and (ii) the choice of a model which can probe the best GM–host interaction with the potential to be translated into the clinic.

## 2. Dysbiosis in Neurological and Psychiatric Disorders

### 2.1. Multiple Sclerosis

Multiple sclerosis (MS) is a chronic neurological disease. According to the World Health Organization (WHO), it affects over 2.8 million people worldwide, predominantly young adult females, with 2.1 new cases per 100,000 people per year. It is characterized by chronic fatigue, reduced quality of life, and disruptions in daily activities. MS is an autoimmune inflammatory disorder leading to demyelination and axonal loss in various regions of the CNS. The disease exhibits significant inter-patient variability and has a complex etiology involving multiple genetic and environmental factors [26]. While MS has been linked to infections by bacteria such as *Staphylococcus aureus* and *Chlamydia pneumoniae*, or viruses like Epstein–Barr [27], recent research has highlighted GM dysbiosis as a crucial feature of the disease.

The idea of targeting GM to alleviate MS symptoms has led to extensive research in the field. Experimental autoimmune encephalomyelitis (EAE) animal models, which mimic MS, exhibit BBB disruption that allows activated leukocytes (macrophages, T and B cells) to enter the CNS, causing further inflammation and demyelination [28]. EAE can be induced by brain peptides from myelin oligodendrocyte glycoprotein (MOG), myelin basic protein (MBP), or proteolipid protein (PLP) combined with inflammation-inducing agents, or by the adoptive transfer of MOG-specific T lymphocytes [29]. EAE models show an increase in microbes that can induce inflammation and disrupt the BBB, and a decrease in microbes that protect the gut barrier and BBB, suggesting a potential role for GM changes in MS onset. Initial studies using antibiotics to alter GM demonstrated that selective microbial reduction could attenuate disease symptoms. These improvements were linked to the actions of invariant natural killer T cells (iNKTs) [30], increased regulatory IL-10-producing Tregs [31], and other immunosuppressive cells such as tolerogenic dendritic cells and anti-inflammatory macrophages [32].

Similarly, our group found that antibiotic administration early in the life of EAE rat models reduces Firmicutes and Actinobacteria, particularly *Helicobacteraceae*, *Spirochaetaceae*, and *Turicibacteriaceae*, while favoring Proteobacteria and Bacteroidetes. This shift leads to an earlier onset of EAE symptoms [33]. Germ-free animals developed milder EAE symptoms with fewer pathological Th1 and Th17 cells and more protective Treg cells compared to conventionally colonized mice [34]. Segmented filamentous bacteria in the small intestine were found to induce Th17 cells that protect against pathogens but can cause inflammation if their numbers are excessive.

Another mechanism by which GM could influence MS is molecular mimicry, where adaptive immune cells mistakenly target autoantigens similar to microbial antigens. The idea of molecular mimicry is based on the cross-reactivity between the host peptide (CNS antigens in the case of MS) and microbial peptides of similar structure. The CNS peptide MBP was found to be similar to some proteins of species belonging to *Bacteroides* and *Bifidobacteria*, and with GDP-L-fucose synthase, the enzyme produced by *Akkermansia* and species of *Prevotella*. This process can trigger inflammation leading to initial host tissue destruction, contributing to autoantigen presentation, antigen spreading, and activation of bystander T cells, further damaging host tissues. Interestingly, ampicillin pretreatment in EAE animals led to the loss of the *Alobaculum* strain (OTU0002) from *Erysipelotrichaceae* and the alleviation of the disease, and monocolonization of germ-free mice with this strain worsened EAE symptoms, but to a lesser extent, suggesting collaboration with other GM members [35]. In silico analysis identified potential molecular mimicry candidates, such as the UvrABC system protein A from *Lactobacillus reuteri* and aminopeptidase from OTU0002. Although these antigens alone did not induce demyelination, clinical scores and incidence were significantly higher when co-colonizing mice than in monocolonized mice, confirming their synergy and suggesting the need for additional microbiota factors for EAE development.

SCFAs also play a role in MS. Oral vancomycin treatment improved EAE by increasing the abundance of SCFA producers like *Clostridium* clusters XIV and XVIII and *Anaerotruncus colihominis*. Our recent research showed a reduction in butyrate-producing bacteria (e.g., *Prevotellaceae*, *Odoribacter*, *Butyricicoccus*, *Ruminococcaceae UCG-008*) and propionate-producing bacteria (e.g., *Muribaculaceae*) in EAE rat models [36]. A reduction in SCFA-producing bacteria is common in EAE and MS patients, with lower levels of *Odoribacter*, *Butyricicoccus* [37], *Prevotella* [38], and *Veillonellaceae* [39] found in patients with more severe symptoms. By inhibiting HDAC (reviewed in [40]) in CD4+ T cells, SCFAs promote IL-10-producing Tregs by stimulating Foxp3 expression and inhibit the production of pro-inflammatory mediators (iNOS, IL-6, TNF-α, IL-1β) in CNS, thus preventing or attenuating autoimmune process development. Interestingly, by the HDAC inhibition in oligodendrocytes, butyrate inhibits demyelination and stimulates remyelination. Additionally, by promoting tight junction protein expression, these metabolites maintain and increase BBB integrity. The recurring association of the MS symptom intensity and immunological profile with lower levels of SCFA-producing bacteria and SCFA concentrations has paved the way for investigating therapies that involve the supplementation of patients with SCFA, SCFA-producing bacteria, or fiber-rich diets favoring these bacteria. One of the most significant studies was performed by Duscha et al. [41]. Based on the hypothesis that changes in SCFA-producing bacteria result in a lower systemic propionic acid concentration, the authors performed a proof-of-concept study in which MS patients were supplemented with propionic acid. In their study, 91 MS patients and 24 healthy controls were supplemented with 1000 mg per day of propionic acid during a period of 14 days, and 52 MS patients continued propionic acid supplementation after this period and were included in a longitudinal study that lasted for 3.34 ± 1.59 months. While the propionic acid supplementation decreased the Treg cell number and function in both patients and healthy controls, the Th17 and Th1 cell numbers decreased only in MS patients after 14 days of supplementation. The immunomodulatory effect was still present after 90 days of supplementation when compared to the basal level detected for each volunteer before the supplementation. Importantly, this Treg/Th17 shift correlated with a reduced annual relapse rate and reduced disease progression in 97 patients who continued supplementation for at least one year.

MS and EAE are also marked by an overgrowth of pro-inflammatory genera such as *Romboutsia*, families *Peptostreptococcaceae*, *Peptococcaceae*, and *Akkermansia muciniphila*, and *Acinetobacter calcoaceticus* [42,43,44,45]. Some of these GM changes are linked to a diet low in plant carbohydrates, which promotes a pro-inflammatory environment and may contribute to autoimmune diseases like MS in genetically predisposed individuals. A carbohydrate-poor diet favors bacteria like *Akkermansia*, which degrades mucin, potentially leading to barrier degradation and inflammation [46]. Further, *Parabacteroides distasonis*, reduced in MS patients [45], has been linked to increased levels of beneficial Tregs in antibiotic-treated or germ-free mice. This bacterium has demonstrated anti-inflammatory effects in rheumatoid arthritis by improving gut barrier function and restoring Th17/Treg balance through the production of secondary bile acids (e.g., lithocholic acid, deoxycholic acid) [47]. The reduction in *Adlercreutzia*, *Prevotella*, *Parabacteroides*, and *Bifidobacterium* in MS patients and EAE animals correlates with their ability to metabolize phytoestrogens from legumes, whole grains, and fruits to monomers [48]. Mice on an isoflavone-rich diet exhibited increased CD8+ T cells in the colon compared to those on an isoflavone-free diet [49]. Given that endogenous estrogen induces Tregs and phytoestrogen metabolites resemble estrogen, these metabolites may induce regulatory CD8+ T cells, mitigating EAE symptoms. Despite the general trend of decreased immunosuppressive or increased pro-inflammatory microorganisms in MS-related microbiota, the variability in taxa makes a universal treatment challenging. Additionally, the culturing of many gut microorganisms remains difficult due to their anaerobic nature and unknown conditions. Consequently, fecal transplantation (FT)—filtering and homogenizing healthy donor stool—has emerged as a promising therapeutic approach (ongoing clinical trial NCT03594487).

Interestingly, microbiota could also contribute to the sex bias in MS, since some sex-specific characteristics of the homeostatic immune system are driven by sex-specific features of microbiota [50]. For example, pregnancy, which is characterized by significant changes in hormonal status and immune system function, coincides with specific changes in microbiota composition and an increased risk of MS development [51]. This hypothesis is supported by the fact that sex-specific microbiota regulate the higher number of T cell precursors in the thymus, the stronger type 1 interferon response in the ileum, and the lower gut population of FoxP3+ Tregs in germ-free male and female rodents, probably through the sex hormones. Thus, all these data together led to the emerging hypothesis in the field that immune-related diseases with sex bias could be associated with sex-specific microbiota [52]. There are at least three different ways in which sex-specific microbiota could modulate the levels of circulating sex hormones: (i) by modulating the gastrointestinal excretion (species that express β-glucuronidase enzymes), (ii) by bioconversion of the endogenous hormones, and (iii) by metabolizing dietary phytoestrogens [53]. Interestingly, the study by Benedek et al. demonstrated that 17β-estradiol administration leads to changes in the microbiota (marked by higher abundances of Lactobacilli) and ameliorates EAE associated with higher Treg abundance in mice [54]. Likewise, it is also shown that estrogen modulates astrocyte function to confer a neuroprotective effect in EAE animal models [55]. Taken together, these findings highlight the association between sex-specific features of the immune system and microbiota in MS as a promising path worth further investigation.

### 2.2. Major Depressive Disorder

Major depressive disorder (MDD) is a mental health disorder characterized by persistent feelings of sadness, hopelessness, and loss of interest in previously enjoyed activities [56]. The WHO estimates that 3.8% of the population experience depression (4% among men, 6% among women), which affects approximately 280 million people globally [57]. It is a multifaceted condition influenced by a complex interplay of genetic, biological, environmental, and psychological factors [58]. While the exact pathophysiology remains elusive, emerging evidence underscores the critical role of neuroinflammation, neurotransmitter imbalances, and neurotrophic factor dysregulation in its development and progression [59]. The GBA has emerged as a promising avenue for understanding the etiology and treatment of depression. A growing body of evidence suggests that alterations in GM composition may contribute to the development of depressive symptoms through various mechanisms, including immune activation, neuroinflammation, and the production of neuroactive metabolites [60]. Various microbiome-derived metabolites are linked to MDD. Dopamine regulates anhedonia, a core symptom of MDD [57], while the availability of tryptophan is associated with mood imbalances [61]. LPS can increase the conversion of tryptophan to kynurenine, reducing central serotonergic availability and producing neurotoxic metabolites like QUIN [19]. The kynurenine pathway links GM with neuroinflammation and depression [19].

Multiple studies reported GM dysbiosis in individuals with MDD, suggesting a potential role of these microbes in the pathophysiology of the disease. Firmicutes, Actinobacteria, and Bacteroidetes are the primary phyla affected, with a large increase in the Bacteroidetes/Firmicutes ratio [62]. While specific bacterial genera, such as *Bacteroides*, are more abundant in these patients, other genera like *Blautia*, *Faecalibacterium*, and *Coprococcus* are less prevalent [30,63,64,65]. However, the variability at the family level significantly increases. Despite the inconsistency between studies, several patterns have emerged. *Prevotellaceae* is consistently reduced in individuals with depression, suggesting its potential role in maintaining gut health [66,67,68]. *Lachnospiraceae* and *Coriobactericaceae* show inconsistent results, being both increased and decreased [65]. Instead, genera *Eggerthella*, *Oscillibacter*, *Holdemania*, *Streptococcus*, and *Desulfovibrio* have been repeatedly found to increase in MDD patients compared to healthy controls [66,68,69,70,71], whereas *Sutterella*, *Faecalibacterium*, *Coprococcus*, and *Clostridium* cluster XIVa are notably reduced in individuals with depression [62,67,72,73,74]. These genera are often associated with anti-inflammatory properties and maintaining gut health, and their reduced presence may contribute to the pro-inflammatory state observed in MDD.

The transfer of fecal microbiota from MDD patients can reduce GM diversity and elevate plasma kynurenine levels in rodents, indicating the transfer of depressive-like traits via microbiota [75], thus corroborating the causal role of GM dysbiosis in MDD. Moreover, the pathological changes associated with depression can exacerbate dysbiosis, creating a vicious cycle that further impacts gut health [76]. Psychological stress can alter GM, and these abnormalities can influence emotional behavior [5]. The depletion of serotonin, dopamine, and GABA is considered a significant risk factor for developing depression [57].

Notably, *Lactobacillus* and *Bifidobacterium* species are decreased in the mouse model of depression [57], suggesting a potential link between reduced GABA production by these bacteria and the development of depressive symptoms. Specifically, *Lactobacillus rhamnosus* JB-1 is able to increase hippocampal GABA receptor mRNA expression and reduce stress-induced corticosterone levels and anxiety- and depression-related behaviors through vagus-nerve-mediated gut–brain axis communication [68]. This highlights its potential to be used as a therapeutic adjunct for depressive disorders. SCFAs play an important role in the pathophysiology of depression [76]. They influence gut health and appetite and may also affect psychological well-being. SCFAs are suggested as potential therapeutic targets in depression [77]. They are frequently depleted in patients with MDD, and their administration, particularly butyrate, has demonstrated antidepressant effects by improving gut permeability and modulating the hypothalamic–pituitary–adrenal (HPA) axis [19]. In addition, acetate can cross the BBB and reduce appetite, while butyrate induces anti-inflammatory effects through regulatory IL-10-producing Tregs, with its deficiency associated with increased depression-like symptoms [78]. The metabolism of bile acids (BAs) is also altered in depression. The lower BA levels produced by gut bacteria correlate with increased severity of depression [79]. Of note, *Turicibacter*, which is involved in BA production, is reduced in depression [79]. In addition, trimethylamine-N-oxide, a metabolite derived from GM, is associated with more severe symptoms of depression [80].

Microbiota-based therapeutics are a promising approach and could be the next breakthrough in the treatment of depression. A total of 178 probiotic species were recently identified as capable of attenuating the depressive phenotype, with *Lactobacillus* spp. and *Bifidobacterium* spp. being the most studied [63]. Liu and colleagues showed that the administration of *Lactobacillus plantarum* PS128 stimulated psychomotor activity in both stressed and naïve adult mice during early life [81]. *L. plantarum* PS128 contributed to a reduction in anxiety behavior in naïve adult mice in a cross-maze test and increased psychomotor activity in both stressed and naïve adult mice in early life in an open field test. The administration of *L. plantarum* PS128 decreased serum corticosterone levels under both primary and stressed conditions, while serum anti-inflammatory cytokines increased in mice that experienced early stress. Probiotic administration alleviated behavioral deficits and emotional memory processing in a myocardial-infarction-induced depression model [82]. The maternal separation test showed that supplementation with probiotics *L. helveticus* R0052 and *L. rhamnosus* R0011 restored normal development and emotional stability in rats experiencing early developmental stress [83]. Tian and colleagues identified the psychobiotic potential of *B. breve* CCFM1025, which reduced anxiety and depressive behaviors, alleviated HPA axis-related inflammation, and upregulated BDNF expression in mice [77]. Similarly, Hao and colleagues demonstrated that *Faecalibacterium prausnitzii* ATCC 27766 supplementation prevented stress-related effects, such as increased corticosterone and IL-6 levels in stressed rats [84]. Human studies have also examined the efficacy of using probiotics to treat depression. Probiotic treatments contained several species of lactobacilli and bifidobacteria, although in one case, *Clostridium butyricum* MIY was added, whereas *St. thermophilus* was added in the other. These probiotic treatments demonstrated significant improvements in depression patients, highlighting the efficacy of different probiotic species in alleviating depressive symptoms [85,86,87,88,89,90,91,92]. An umbrella meta-analysis revealed that probiotic supplementation significantly reduced depression symptoms, particularly in studies where the intervention lasted more than eight weeks and the dosage exceeded 10 × 10^9^ colony-forming units (CFU) [93].

### 2.3. Alzheimer’s Disease

Alzheimer’s disease (AD) is a neurodegenerative disorder characterized by progressive neuronal loss and cognitive impairment. Based on the data from WHO, more than 55 million people currently have dementia worldwide, with nearly 10 million new cases every year. The primary hallmarks of AD include the accumulation of amyloid beta (Aβ) peptides, tau aggregates, and neuroinflammation [94]. AD is classified into two main types: late-onset (sporadic) and early-onset (familial AD, FAD). FAD is typically caused by mutations in genes related to Aβ generation, including amyloid beta precursor protein (APP), presenilin 1 (PS1), and presenilin 2 (PS2) [95]. Sporadic AD, on the other hand, is mainly influenced by aging, genetic predisposition (with apolipoprotein E (APOE) contributing to approximately 60–80% of cases), and environmental factors such as diet, sleep, and exercise [96].

Recent research has highlighted the significant role of GM in AD, particularly how age-related perturbations in GM can impact disease development [97]. GM dysbiosis is associated with increased BBB permeability, which may contribute to a pro-AD microenvironment [98]. Metagenomic studies have reported dysbiosis in AD patients compared to elderly individuals without AD [99]. Notably, GM dysbiosis is also observed in cognitively normal individuals with biomarker evidence of AD, such as Aβ-positive status (preclinical AD) [100]. This suggests that GM changes may occur before the symptomatic onset of AD.

In preclinical AD, the Bacteroidetes/Firmicutes ratio remains similar to that in healthy individuals, but there is an increased abundance of *Bacteroides* species (e.g., *Bacteroides intestinalis* and *Bacteroides caccae*), which may serve as potential biomarkers for AD onset. Symptomatic AD patients show a significant increase in Bacteroidetes and a decrease in Firmicutes [99] and Actinobacteria [101]. Specifically, AD patients exhibit an increase in the families *Bacteroidaceae* and *Rikenellaceae* and a decrease in *Ruminococcaceae*, *Turicibacteraceae*, *Peptostreptococcaceae*, *Clostridiaceae*, and *Mogibacteriaceae* [102]. The imbalance among these dominant phyla of anaerobic bacteria is also seen in other chronic inflammatory conditions. These bacteria ferment dietary fibers into SCFAs, which play a role in gut–brain communication [102]. In AD patients, shifts in GM composition correlate with lower fecal SCFA levels [103].

Similar patterns of GM changes have been observed in various transgenic mouse models of AD, including decreases in Firmicutes and increases in Bacteroidetes [104]. Additionally, *Ruminococcus* sp. (family *Ruminococcaceae*) and *Butyricicoccus* sp. (family *Clostridiaceae*) are significantly reduced in these models, along with lower SCFA levels in feces and the brain [105]. FT experiments further support the role of GM in AD. Transplantation of GM from AD-prone mice impaired memory function in healthy recipient mice, and this cognitive decline could be partially rescued by eliminating the GM from AD mice with antibiotics [106,107].

Emerging evidence suggests potential therapeutic effects of SCFAs in AD. For example, acetate inhibits Aβ aggregation in vitro, while butyrate influences microglial maturation and negatively correlates with Aβ pathology [108]. Additionally, elevated serum levels of secondary BAs (e.g., deoxycholic, glycodeoxycholic, and taurolithocholic acids) are positively correlated with cognitive decline in AD patients, and high levels of these BAs are associated with pro-inflammatory bacteria such as *Alistipes* sp. and *Bacteroides* sp. [109]. Tryptophan metabolites, particularly indole-3-propionic acid, have shown potential in preventing the development of Aβ fibrils, indicating their possible therapeutic role in AD [110].

Several animal models are used to study host–microbe interactions in AD. AD mouse models with FAD-related APP and PS1 mutations have been used to test probiotics from the *Lactobacillus* and *Bifidobacteria* genera. For example, the probiotic *Bifidobacterium breve* prevented memory impairment and reduced Aβ production and microglia activation in the APP knock-in mouse AD model [111]. Interestingly, AD can also be effectively modeled in the nematode *Caenorhabditis elegans*. This versatile model organism is used to study neurodegenerative alterations and offers a cost-effective alternative to mice for high-throughput drug screening. Transgenic *C. elegans* lines expressing human Aβ peptide and tau protein exhibit clear neurodegenerative phenotypes, including deficits in chemotaxis, associative learning, and locomotion. Research using these AD-like *C. elegans* models has demonstrated the probiotic potential of *Bacillus subtilis*, which can delay neurodegeneration and suppress behavioral impairments [112].

### 2.4. Autism Spectrum Disorders

Autism spectrum disorders (ASDs) comprise a heterogeneous group of neurodevelopmental disorders characterized by impaired social interactions and restrictive and repetitive behaviors. The current estimate of ASD prevalence ranges from 1 in 100 children according to the WHO to 1 in 44 children [113]. The rapid increase in the frequency of ASDs is likely due to improvements in diagnostic criteria and increased public awareness. At present, there are no biomarkers for the diagnosis of ASDs. The heterogeneity of ASD genotypes and phenotypes represents a major hurdle in investigating ASD. The most frequent comorbidities are anxiety, depression, sleep problems, epilepsy, intellectual disability, and GI problems [114]. The most common GI problems include chronic constipation, diarrhea, abdominal pain, and gastroesophageal reflux disease [115]. Although GI problems co-occur with symptoms such as anxiety, sleep issues, and behavioral alterations including aggressive, repetitive, and self-harming behaviors [116], no causative link has been established so far.

As already stated above, lifestyle, particularly diet, is a major contributing factor to GM. The diet of individuals with ASDs usually lacks diversity, which significantly affects GM diversity. Thus, it is not surprising that dysbiosis has been repeatedly found in patients with ASDs. However, more consensus about differences in bacterial abundance has yet to be reached. The first thorough meta-analysis of 18 different studies, including 493 ASD children and 404 controls, was performed by Iglesias-Vazquez et al. in 2020, using Review Manager (RevMan)5.3 [117]. They found an increased abundance of the phyla Bacteroidetes, Firmicutes, and Actinobacteria in the children with ASD, with a significantly higher abundance of the genera *Bacteroides*, *Parabacteroides*, *Clostridium*, *Faecalibacterium*, and *Phascolarctobacterium*, and with a decrease in *Coprococcus* and *Bifidobacterium*. Yang et al. also used RevMan5.3 to analyze data from 28 studies from PubMed, PsycINFO, Web of Science, Scopus, and MEDLINE (up to February 2024), involving 1256 children with ASDs and 1042 neurotypical children [118]. Their study revealed an increase in *Parabacteroides*, *Anaerostipes*, *Faecalibacterium*, *Clostridium*, *Dorea*, *Phascolarctobacterium*, *Lachnoclostridium*, *Catenibacterium*, and *Collinsella*, along with a decrease in *Barnesiella*, *Odoribacter*, *Paraprevotella*, *Blautia*, *Turicibacter*, *Lachnospira*, *Pseudomonas*, *Parasutterella*, *Haemophilus*, and *Bifidobacterium*. Discrepancies were found between studies for *Faecalibacterium*, *Clostridium*, *Dorea*, *Phascolarctobacterium*, *Catenibacterium*, *Odoribacter*, and *Bifidobacterium*, even when excluding individual studies. However, given the complexity of microbiome analysis, difficulties in the interpretation of the results, and the lack of reproducibility, machine-learning-based approaches have started to gain more attention in the GM analysis of ASD individuals with the hope of identifying a specific subset of bacteria that is a reproducible signature for ASD classification. For example, Morton JT et al. developed a Bayesian differential ranking algorithm to uncover ASD-associated molecular and taxa signatures across 10 microbiome datasets and 15 associated datasets, including dietary patterns, metabolomics, cytokine profiles, and brain gene expression [119]. They found a strong ASD-associated GM signature when compared to age- and sex-matched controls, which was not present when compared to siblings. A total of 591 microbes were more commonly found in ASD children, whereas 169 were found in their control counterparts. As with other meta-analyses, greater within-study classification performance than across-study classification performance was found, likely due to microbial heterogeneity across diverse human populations and ASD heterogeneity. Cross-omics analysis revealed an association between diet, microbiome metabolism, host cytokine signatures, and the human brain metabolism and transcriptome, suggesting cross-talk along the GBA in ASDs. For example, *P. copri* depletion and a concurrent decrease in carbohydrates and upregulation of IL-6 were observed in ASD individuals, along with a decrease in *B. thetaiotaomicron* and the depletion of TGF-beta. Peralta-Marzal et al. also applied machine learning, but they used recursive ensemble feature selection (REFS) to analyze 16S rRNA gene sequencing data from 117 subjects (60 ASD cases and 57 siblings) [120]. They identified 26 bacterial taxa that discriminate ASD cases from controls when using neurotypical siblings as controls, for individuals 2 to 7 years old. The validation was performed on two unrelated datasets (125 ASD cases and 98 controls), of which one used siblings as controls and the other used unrelated age-matched children. Thus, machine learning enabled the authors to reduce the relevant ASD features from 2040 amplicon sequence variants (ASVs) to 26 ASVs that were validated in another two datasets, confirming their predictive value. Should this set of bacteria associated with ASDs be confirmed in the independent studies, it could be considered as a potential diagnostic tool for ASDs and used to provide further insights into plausible molecular mechanisms of the GBA in ASD. Specifically, this approach highlighted genera *Bifidobacterium* and *Collinsella* (*Actinobacteria phylum*) to be decreased, whereas *Prevotellaceae* and *Parabacteroides* (Firmicutes phylum) were increased in ASD individuals compared to controls. Within the phylum Firmicutes, *Erysipelatoclostridiaceae*, *Murdochiella*, *Butyricicoccus*, *Clostridium*, *Lachnospiraceae* UCG-004, and *Eubacterium* eligens were decreased, while *Lachnospiraceae*, *Clostridium*, *Sarcina*, *Anaerosporobacter*, and Oscillospira were increased. Additionally, some *Lachnospiraceae* and *Oscillospira* were not present in any control subjects.

To support the role of GM in ASDs, the authors reanalyzed data from a 2-year open-label fecal transplant study involving 18 children with ASDs [121]. For FT, the children underwent a 2-week antibiotic treatment and bowel cleanse, followed by 2 days of high-dose FT treatment and 8 weeks of daily maintenance. Based on the Childhood Autism Rating Scale (CARS), both GI and ASD symptoms greatly improved following the 10-week course of treatment. The improvement was stable after 2 months, with further improvement observed after 2 years in most patients. In addition to the increase in *Prevotella* sp. found in both the original study and by Morton and colleagues, the authors also found an increase in *Desulfovibrio piger*. They also found that 305 taxa remained stable over 2 years, including 13 taxa belonging to the *Prevotella*, *Bifidobacterium*, *Bacteroides*, and *Desulfovibrio* lineages, including *B. fragilis*, *B. thetaiotaomicron*, *B. longum*, and *P. copri* with immunomodulatory potential, and butyrate producers *Butyricimonas* and *Anaerobutyricum* genera, indicating a potential role in the GBA. Therefore, the FT by Kang et al. led to a sustained increase in the total diversity and abundance of bacteria, positively attracting additional symbiotic bacteria to alter the gut ecology and greatly reducing the symptoms of autistic individuals [122]. However, the FT clinical trial performed by Li and colleagues, involving 40 ASD children during a 4-week FT treatment phase and an 8-week follow-up observation phase after the treatment, showed no statistically significant difference in the diversity of GM after the treatment [123]. However, they found that a high abundance of *Eubacterium coprostanoligenes* in ASD patients before FT decreased in some patients (responders to FT). Despite the lack of a cure for ASDs and the U.S. Food and Drug Administration (FDA) “fast-track” label for FT as an ASD treatment following the promising results of Kang and colleagues, the variability in results between FT clinical trials calls for standardized and rigorously designed randomized double-blind placebo-controlled trials to establish the efficacy and safety of FT before considering it as a treatment for ASDs.

Although probiotics are promising interventions for ASDs, clinical trials conducted so far have not reached solid conclusions about their efficacy. Different trials have used various bacterial strains, mainly combinations of *Bifidobacterium longum*, *Bifidobacterium infantis*, *Lactobacillus acidophilus*, *Lactobacillus rhamnosus*, *Lactobacillus plantarum*, *Lactobacillus reuteri*, *Enterococcus faecalis*, and *Streptococcus thermophilus*. Additionally, the trials varied in design, with poor patient stratification and frequent methodological errors (e.g., improper handling of statistics related to multiple comparisons). Despite the frequent observation of no statistical difference between ASD individuals and the control group, probiotics have consistently ameliorated GI and often different behavior-related symptoms in ASD individuals (for a detailed review, see [124]), highlighting their potential despite the limitations of the studies performed thus far.

For example, Santocchi and colleagues conducted a double-blind randomized, placebo-controlled clinical trial with De Simone Formulation probiotics (marketed as Vivomixx^®^ in the EU (Mendes SA, Lugano, Switzerland) and Visbiome^®^ (ExeGi Pharma LLC, Rockville, MD) in the USA; containing 450 billion of eight probiotic strains: *Streptococcus thermophilus*, *Bifidobacterium breve*, *Bifidobacterium longum*, *Bifidobacterium infantis*, *Lactobacillus acidophilus*, *Lactobacillus plantarum*, *Lactobacillus paracasei*, and *Lactobacillus delbrueckii* subsp. *bulgaricus*) on 42 ASD children and 43 placebo controls for six months [125]. No differences between groups were detected using the Total Autism Diagnostic Observation Schedule–Calibrated Severity Score (ADOS-CSS). However, when participants were divided into subgroups with GI symptoms (n = 30) and without GI symptoms (n = 55), the group without GI symptoms showed a significant decrease in ADOS scores compared to the placebo group, with a mean reduction of 0.81 in Total ADOS CSS and 1.14 in Social-Affect ADOS CSS over six months. The group with GI symptoms showed greater improvements in some GI symptoms, adaptive functioning, and sensory profiles compared to the GI group treated with a placebo. A follow-up randomized 6-month controlled trial with 46 children with ASD (18–72 months) using the same probiotics and electroencephalography as an outcome revealed a decrease in power in frontopolar regions in the beta and gamma bands and increased coherence in the same bands, along with a shift in frontal asymmetry. This suggested a modification toward more typical brain activity [126]. Additionally, a significant negative correlation was found between frontopolar coherence in the gamma band and TNF-α (r = −0.30, *p* = 0.04), suggesting that probiotics may modulate ASD-associated neuroinflammation.

## 3. GM–Host Interaction: Future Directions of the Field

### 3.1. Culturomics

In recent decades, probiotic research has significantly expanded. Probiotics are defined as “live microorganisms that, when administered in adequate amounts, confer a health benefit on the host” [127]. There is an increasing body of literature on the mechanisms of probiotic activity, particularly regarding LAB [128,129,130]. The beneficial effects of probiotics are diverse. They provide competitive exclusion of pathogens, improve intestinal barrier function, and modulate the immune system [131,132,133]. Furthermore, probiotics can ameliorate the effects of intoxications caused by heavy metals or cigarette smoke [134,135,136], alleviate symptoms of diabetes [137,138], and even promote longevity and extend lifespan [139,140]. However, despite their great promise, the mechanisms underlying GM–host interactions still need to be elucidated before probiotics can be used as therapeutics or adjunct therapies for neurological and psychiatric disorders. The complexity of GM–host interactions is twofold: (i) many human gut bacteria associated with brain disorders are strict anaerobes (e.g., SCFAs producers) or have unknown cultivation conditions, and (ii) there is an absence of human models that can best probe GM–host interactions with potential for clinical translation. Metagenomics has uncovered the immense diversity of the human GM, with most species remaining uncultured due to unknown or difficult culture conditions. Culturomics, a culturing approach that combines multiple culture conditions, was developed to enable the isolation, cultivation, and sequencing of bacterial species [141]. This approach has revolutionized the cultivation of gut anaerobes, dramatically increasing the number of cultivated strains in pure culture and solving the culturing of many “fastidious” bacteria that require specific nutrients in the culturing media. However, a considerable number of gut bacteria remain uncultivated, necessitating further optimization of culture conditions [142].

### 3.2. Human Models

Another layer of complexity in studying GM–host interactions is the choice of the appropriate model, especially when examining neurological and psychiatric disorders. Animal models, particularly mice, play a pivotal role in translational neuroscience, despite the persistent issue of low translatability observed over decades [143]. The reasons for this low translatability are numerous. In the context of brain disorders, interspecies differences at molecular and anatomical levels, as well as distinct physiology and behavior, play a significant role. Additionally, our limited understanding of the etiology of complex brain disorders, their polygenic nature, and the impact of the environment and microbiota on disease onset, development, and maintenance all contribute to the misuse and overestimation of various animal models. Typically, these models represent only one symptom or endophenotype rather than capturing the full complexity of disorders, leading to the low translatability of the results obtained.

Despite these challenges, mice remain the preferred models for studying human brain disorders. One of the most important reasons is the BBB, a selective semi-permeable membrane that separates the blood from the brain. In brain microvessels, endothelial cells form extensive tight junctions and, together with specific receptors, transporters, efflux pumps, and other cellular components, create a barrier that regulates the exchange of molecules and ions between the vascular compartment and the brain [144]. The BBB is composed of specialized human brain microvascular endothelial cells (HBMECs), perivascular cells (pericytes), and astrocytes [145]. The complete neurovascular unit (NVU) also includes neurons and microglia, which contribute to BBB development and maintenance. When intact, the BBB impedes the influx of most substances from the blood into the brain, including metabolites and drugs. Consequently, while protecting the brain, the BBB almost completely prevents the entry of drugs into the brain, making the pharmacotherapy of brain diseases very challenging.

The field of in vitro models that mimic multicellular organs or cross-talk between different organs is still developing. These models are generally divided into static models, which use transwell systems, and dynamic models, which use microfluidic technology and are also known as Organ-on-a-Chip (OoC) systems. Initially, these models were populated with non-human or immortalized cell lines that were readily available, inexpensive, and easy to manipulate. However, with technological advancements, the field has shifted toward using primary human cells and patient-specific induced pluripotent stem cell (iPSC)-derived cells and organoids (2D and 3D models, respectively), which retain an individual genetic makeup, thus allowing for studying specific phenotypes in the “real-world” genetic background as well as the effect of inter-human variability. These advanced models are now the preferred choice for drug screening and preclinical investigations, providing an efficient alternative to animal models.

Patient-specific iPSC-derived induced neurons (iNeurons) and brain organoids have revolutionized the study of brain disorders by allowing their modeling in a human context and revealing human-specific underlying molecular mechanisms, such as those seen in 7q11.23 copy number variation (CNV)-related disorders [146,147]. Additionally, patient-derived iNeurons and brain organoids have been successfully used for drug screening [142]. However, iNeurons lack the complexity of the brain, and both iNeurons and brain organoids are missing the BBB and blood vessels required for delivering oxygen, nutrients, and metabolites. Consequently, current efforts are focused on integrating brain models with BBB transwell or OoC systems, leading to significant improvements and narrowing the gap with clinics.

In the context of probiotics and the GBA, there are two additional levels of complexity: human-specific GM and the communication between the brain and the gut. Thus, first, the novel, human-specific, GM has to be cultured in vitro, and second, microbiota-derived metabolites and other signaling molecules must traverse both the gut barrier and the BBB, making GBA modeling particularly challenging. Reflecting on George E. P. Box’s quotation, “All models are wrong, but some are useful”, we will describe the state-of-the-art transwell-based and OoC models currently used to study the gut barrier, BBB, and GBA (for an extensive review on this topic, see [25]).

From a technical perspective, the transwell system is cost-effective and relatively easy to set up and control and offers various endpoints, including trans-epithelial/endothelial electrical resistance (TEER) measurements and biochemical assays [145]. On the other hand, microfluidic-based OoC technology represents a sophisticated cell culture system with controlled flow and velocity that mimics the physicochemical microenvironment [148]. This system is considered the most physiologically relevant human model available today. The shear stress provided by continuous flow in microfluidic systems is crucial for enhancing cell longevity, positively influencing cell differentiation and maturation, regulating gut and BBB transport, and preventing de-differentiation [145]. Thus, the continuous flow enabled by microfluidic systems offers a significant advantage for in vitro modeling of gut and BBB functions and aids in translating obtained results into clinical applications.

#### 3.2.1. The Gut Barrier

Caco-2 cells, an immortalized cell line derived from human colorectal adenocarcinoma, are considered the gold standard for intestinal models [149]. These cells spontaneously proliferate, differentiate, and form intercellular tight junctions. When cultured in a monolayer, Caco-2 cells provide a physical and biochemical barrier for ions and small molecules, making them widely used for ADME-Tox (adsorption, distribution, metabolism, excretion, and toxicology) studies [150].

**Figure 2:**
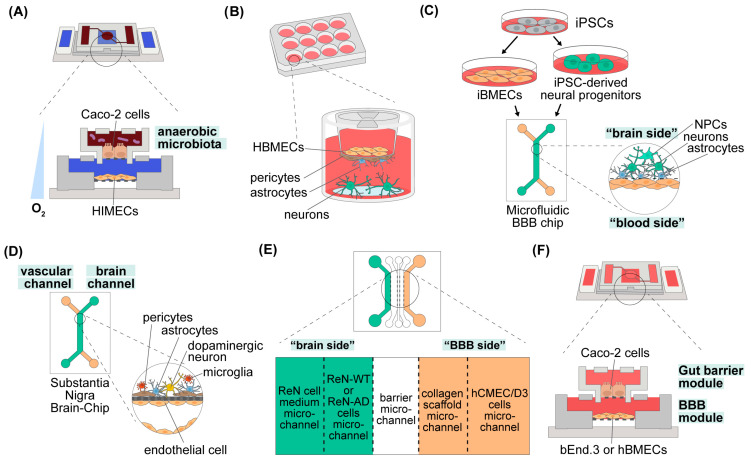
An overview of the state-of-the-art models used to study GM–host interactions. Schematic representations of the following models are presented: (**A**) gut barrier (intestine-on-a-chip, Intestine Chip) [153]; (**B**) static BBB model composed of astrocytes, pericytes, brain microvascular endothelial cells (HBMECs) and neurons [145]; (**C**) human iPSC-derived BBB-on-a-Chip model composed of induced BMECs (iBMECs), induced astrocytes (iAstrocytes), and iNeurons [155]; (**D**) “The Substantia Nigra Brain-Chip”, composed of human iBMECs, pericytes, astrocytes, microglia, and induced dopaminergic neurons [156]; (**E**) AD-BBB model, in which the brain side is composed of human neural progenitor ReN cells, wild type (ReN-WT) or expressing familial AD-related APP and APP/PSEN1 mutations (ReN-AD models), whereas the BBB side is composed of the brain microvascular endothelial cell line hCMEC/D3 [158]; (**F**) GBA-on-a-Chip model composed of the gut barrier (Caco-2 cells) and the BBB barrier (populated with murine brain endothelial cell line bEnd.3 or hBMECs) [157].

To enhance the Caco-2 model further, various microfluidic systems incorporating fluid shear stress similar to physiological conditions have been developed [150,151,152]. One of the most advanced Caco-2-based microfluidic systems, which has made significant strides in the field of GM–host interactions, is the intestine-on-a-chip (Intestine Chip). This model supports interactions between the human intestinal epithelium and both aerobic and anaerobic commensal gut microorganisms in a complex environment (Figure 2, panel (A), [153]). The Intestine Chip incorporates a hypoxia gradient across the engineered endothelium–epithelium interface, enabling the co-culturing of complex anaerobic and aerobic human commensal gut bacteria within the same channel as the mucus-producing human villus intestinal epithelium. The chip builds on a previously published two-channel microfluidic Organ Chip device made from poly-dimethylsiloxane (PDMS) polymer, which features upper and lower microchannels separated by a porous membrane [151]. In this setup, the upper channel contains Caco-2 cells in direct contact with human GM, while the lower channel is lined with human intestinal microvascular endothelial cells (HIMECs). The chips are placed in an anaerobic chamber to create a physiologically relevant oxygen gradient similar to that found in the human intestinal epithelium and microvascular endothelium. Microscale oxygen sensors, based on oxygen-quenched fluorescent particles, are integrated into the chip to measure oxygen levels in situ. To maintain conditions suitable for anaerobic microbiota growth, the upper anaerobic chamber is continuously flushed with humidified 5% CO_2_ in nitrogen gas. Simultaneously, small amounts of oxygen diffuse from the bottom channel through the porous PDMS membrane from the well-oxygenated medium flowing through the lower endothelium-lined channel. This arrangement provides sufficient oxygen to maintain Caco-2 cells at the bottom of the anaerobic channel, establishing a functional host–microbiome interface. Under this hypoxia gradient, Caco-2 cells differentiate and form tight junctions, and HIMECs form a monolayer. Notably, in the presence of obligate anaerobes, the barrier function is enhanced rather than diminished compared to aerobic conditions. The barrier remains functional for at least 8 days in culture. The physiologically relevant low-oxygen microenvironment on-chip results in a higher level of microbial diversity (~200 unique operational taxonomic units (OTUs)) and an increased abundance of obligate anaerobic microbiota, thus maintaining microbial diversity similar to that of the human gut.

#### 3.2.2. The Blood–Brain Barrier

Initial models of the BBB relied on animal BMECs, but species-specific differences limit their utility. Consequently, the field shifted toward isolating primary human BMECs and generating immortalized lines. Although widely used, these models show some limitations, primarily in their barrier properties [154]. Currently, the best BMECs are iPSC-derived. These cells exhibit proper organization of tight junctions and appropriate expression of nutrient and efflux transporters, and importantly, they form an effective barrier as measured by TEER, with drug permeabilities that highly correlate with in vivo measurements. However, despite these advantages, transcriptomic data from iPSC-derived BMECs (iBMECs) indicate that these cells also express several epithelial markers in addition to endothelial ones [154]. Thus, further optimization of the protocol for iBMEC differentiation is needed.

Over the years, various two- and three-cell BBB models using transwells have been developed. Stone and colleagues developed a BBB model composed of four primary human cell types: astrocytes, pericytes, HBMECs, and neurons (Figure 2, panel (B), [145]). In this model, astrocytes and pericytes were seeded on the basolateral side of the insert, HBMECs on the apical side, and neurons on a poly-L-lysine-coated coverslip on the plate bottom. This setup allows TEER measurements without disturbing any cell type. The mixed co-culture of astrocytes and pericytes proved to be more stable and significantly increased TEER compared to separate cultures. This model, derived from four primary human cells, is the closest to the human NVU among all other static BBB models.

Conversely, Vatine and colleagues developed a human iPSC-derived BBB-on-a-Chip composed of iBMECs, induced astrocytes (iAstrocytes), and iNeurons (Figure 2, panel (C) [155]). Using isogenic iPSC lines (lines with the same genetic background), they derived both iBMECs and a mixed culture of iNeurons and iAstrocytes. The chip, made from PDMS, consists of two opposed, parallel microchannels separated by a porous flexible membrane. iBMECs were seeded on the bottom channel to represent the blood side, while iPSC-derived neural progenitors were seeded on the brain side and differentiated into a mixed neural culture containing progenitors, astrocytes, and mature neurons. Calcium imaging showed spontaneous neuronal activity that could be blocked by tetrodotoxin (TTX), indicating electrophysiological activity. iAstrocytes on the brain side extended processes through the porous membrane to form direct cell-to-cell contact with iBMECs on the blood side, creating a functional BBB. The permeability for dextran-FITC was lower when iBMECs were co-cultured with iNeurons/iAstrocytes than when cultured alone, demonstrating that this co-culture supports iBMEC functional maturation even in the absence of pericytes. A specially engineered version of the BBB-Chip with incorporated gold electrodes on both sides of the porous membrane measured TEER, which reached 1500 Ω × cm^2^ two days post-seeding and remained above 1000 Ω × cm^2^ for five days, indicating physiologically relevant values.

Similarly, Pediaditakis and colleagues developed “The Substantia Nigra Brain-Chip”, which includes human iBMECs, pericytes, astrocytes, microglia, and induced dopaminergic neurons, to study alpha-synuclein (αSyn) aggregation in Parkinson’s disease (PD; Figure 2, panel (D) [156]). This chip, similar to the one created by Vatine et al. [155], consists of two microfluidic channels fabricated from PDMS and separated by a porous PDMS membrane. The brain microchannel was populated with iPSC-derived dopaminergic neurons and human primary pericytes, astrocytes, and microglia, while the vascular channel was lined with iBMECs. Dopamine levels were significantly higher in the chip compared to mono-cultured iPSC-derived dopaminergic neurons, highlighting the impact of a physiologically relevant microenvironment on neuron maturation. The model reproduced in vivo-relevant synucleinopathies upon exposure to αSyn fibrils, showing progressive accumulation of phosphorylated αSyn, mitochondrial dysfunction, oxidative stress, neuroinflammation (microglia activation and astrogliosis), compromised barrier function, and a time-dependent neuronal loss, all the phenotypes described in PD patients and the relevant animal models [156]. “The Substantia Nigra Brain-Chip” model emphasized the importance of relevant mechanical forces and cytoarchitecture (including cell–cell interactions) for brain disease models’ maturation and function. It effectively replicated PD patients’ endophenotypes and demonstrated the model’s validity in studying brain disease mechanisms, including BBB dysfunction and relevant drug treatments.

#### 3.2.3. The Gut–Brain Axis

As mentioned above, the GBA is a bidirectional communication network between the gut and brain, involving neuronal, endocrine, metabolic, and immune signaling molecules that must pass through two semi-permeable barriers: the gut barrier and the BBB [25]. Modeling the GBA presents a challenge due to the complexity of communication between the gut and the brain, two distinct and distant organs. To our knowledge, Kim and colleagues recently reported the first and only GBA-on-a-Chip model (Figure 2, panel (F) [157]). They created a modular microfluidic chip where the gut barrier module (populated with the human gut epithelial Caco-2 cells) was in the upper part, and the BBB module (populated with murine brain endothelial cell line bEnd.3 or hBMECs) was in the lower part. The upper and lower parts were interconnected via microfluidic channels. Both modules formed functional barriers when cultured appropriately. The functionality of the barriers was assessed by challenging them with pro-inflammatory LPS and butyrate, one of the SCFAs known to have a host-beneficial effect. As expected, exposure to LPS decreased TEER values and increased permeability of both membranes, with condition-dependent variability. Conversely, treatment with butyrate improved the integrity of both barriers. Although simple, this GBA model provides a proof of concept that both the gut and blood–brain barriers can be modulated by various bacterial products and metabolites, affecting permeability and consequently the influx and efflux of various ions and molecules into and out of the brain. Currently, at least three large collaborative projects are aiming to create complex GBA models: the MINERVA project (ERC Grant agreement No. 7247341, https://cordis.europa.eu/project/id/724734, accessed on 8 August 2024), the IMBIBE project (ERC Grant agreement No. 723951, https://cordis.europa.eu/project/id/723951, accessed on 8 August 2024), and GUTVIBRATIONS (ERC Grant agreement No. DT-NMBP-23, under the Next-Generation Organ-On-Chip Call, https://gutvibrations.org/gutvibrations/, accessed on 8 August 2024). While we await results from these collaborative efforts, combining existing Gut-on-Chip and BBB-on-Chip technologies could significantly advance the field of probiotics, especially for psychobiotics.

## 4. Conclusions

To model GM–host interactions and the polygenic nature of the discussed disorders, alongside inter-human variability, the field is shifting towards iPSC lines and their derivatives as the cornerstone of personalized medicine. Indeed, being patient-derived, iPSC lines on the one hand resemble all the complexity of the genetic makeup of diverse neuropsychiatric disorders, and on the other hand, they capture inter-human variability. Shifting from animal models to a human iPSC-based disease modeling approach, which reflects individual patient characteristics, should streamline the translation to the clinic. However, concerns have been raised about using iPSC lines for modeling neurodegenerative disorders due to the erasure of epigenetic marks. A recent study by Ng et al. helps to address this concern. Ng and colleagues compared iPSC-derived glutamatergic neurons from thirteen sporadic AD patients and one FAD patient with patient data and postmortem samples. They demonstrated that sporadic-AD-patient-derived iPSC models can reproduce the functional consequences of increased Aβ burden [159]. Specifically, they showed that Aβ levels in the media negatively correlated with Aβ levels from patient donors. Exogenous Aβ insults led to synapse loss that reflected patient-specific vulnerability but showed consistency across different differentiation rounds. Additionally, patient cognitive decline correlated with individual levels of Aβ burden, which was consistent with synapse loss in iNeurons caused by Aβ insults in vitro, reflecting in vivo vulnerability to Aβ for both sporadic and familial AD patients.

While iPSC-based models for MDD are still in their early stages, and the ASD field is focusing on patient-derived iNeurons and brain organoids, existing models include the BBB for MS and AD. The MS-BBB model involves the co-cultivation of MS-derived iPSC-based endothelial cells, pericytes, and astrocytes [160,161,162]. The AD-BBB model comprises a 3D human neural model and a brain endothelial cell monolayer with a BBB-like phenotype on a chip (Figure 2, panel (E) [158]). ReN cells, commercially available human neural progenitor cells, were used for the brain side, with ReN wild-type (WT) cells as controls and ReN cells expressing familial AD-related APP mutations (ReN-GA) and APP/PSEN1 mutations (ReN-mGAP) as AD models. For the BBB side, hCMEC/D3 cells were used. Both static and dynamic BBB models demonstrated disease-dependent alterations in BBB permeability. It would be valuable to use these BBB models, alone or in combination with gut barrier modules, to gain mechanistic insights into how gut bacteria modulate neurological and psychiatric disorders discussed in this review. Preliminary evidence of the direct effects of GM members and their metabolites on neurons comes from Gisevious and colleagues, who showed that propionate and butyrate promote neurite recovery in MS-patient-derived iNeurons [163]. This effect was associated with extensive changes in chromatin assembly through the inhibition of histone deacetylase class I/II and alterations in translational and metabolic processes.

In conclusion, the relationship between GM and mental health has garnered significant attention in recent years. Probiotics, as modulators of GM, represent a promising avenue for developing novel therapeutic strategies. While preliminary studies suggest potential benefits in alleviating symptoms of the neurological and psychiatric disorders discussed in this review, further research is essential to elucidate underlying mechanisms and identify optimal probiotic strains for specific patient populations. A multidisciplinary approach, including clinical trials, mechanistic studies with the models discussed, and translational research, is crucial for unlocking the full potential of probiotics in managing these disorders.

## Abbreviations

Gut microbiota	GM
Induced pluripotent stem cells	iPSCs
Short-chain fatty acids	SCFAs
Histone deacetylase	HDAC
G-coupled protein receptors	GPRs
5-hydroxytryptamine	5-HT
Central nervous system	CNS
Gastrointestinal	GI
Blood–brain barrier	BBB
Kynurenic acid	KYNA
Quinolinic acid	QUIN
N-methyl D-aspartate	NMDA
Indoleamine-2,3-dioxygenase	IDO
γ-amino butyric acid	GABA
Lactic acid bacteria	LAB
Glutamate (glutamic acid) decarboxylase	GAD
Gut–brain axis	GBA
Lipopolysaccharides	LPSs
T and B lymphocytes	Treg and Breg cells
Enteric nervous system	ENS
Multiple sclerosis	MS
Encephalomyelitis	EAE
Myelin oligodendrocyte glycoprotein	MOG
Myelin basic protein	MBP
Proteolipid protein	PLP
Invariant natural killer T cells	iNKTs
Fecal transplantation	FT
Major depressive disorder	MDD
World Health Organization	WHO
Hypothalamic–pituitary–adrenal	HPA
Bile acids	BAs
Colony-forming units	CFU
Alzheimer’s Disease	AD
Amyloid beta	Aβ
Familial AD	FAD
Amyloid beta precursor protein	APP
Presenilin	PS
Apolipoprotein E	APOE
Autism Spectrum Disorders	ASDs
Childhood Autism Rating Scale	CARS
Food and Drug Administration	FDA
Total Autism Diagnostic Observation Schedule–Calibrated Severity Score	ADOS-CSS
Human brain microvascular endothelial cells	HBMECs
Neurovascular unit	NVU
Organ-on-a-Chip	OoC
Induced neurons	iNeurons
Trans-epithelial/endothelial electrical resistance	TEER
ADME-Tox	adsorption, distribution, metabolism, excretion, and toxicology
Poly-dimethylsiloxane	PDMS
Human intestinal microvascular endothelial cells	HIMECs
Operational taxonomic units	OTUs
iPSC-derived BMECs	iBMECs
Induced astrocytes	iAstrocytes
Tetrodotoxin	TTX
Parkinson’s disease	PD
Review Manager	RevMan
Recursive ensemble feature selection	REFS
Amplicon sequence variants	ASVs

## References

[1] Mizrahi-Man O., Davenport E.R., Gilad Y. (2013). Taxonomic classification of bacterial 16S rRNA genes using short sequencing reads: Evaluation of effective study designs. PLoS ONE.

[2] Hugon P., Dufour J.C., Colson P., Fournier P.E., Sallah K., Raoult D. (2015). A comprehensive repertoire of prokaryotic species identified in human beings. Lancet Infect. Dis..

[3] Li J., Jia H., Cai X., Zhong H., Feng Q., Sunagawa S., Arumugam M., Kultima J.R., Prifti E., Nielsen T. (2014). An integrated catalog of reference genes in the human gut microbiome. Nat. Biotechnol..

[4] Rodriguez J.M., Murphy K., Stanton C., Ross R.P., Kober O.I., Juge N., Avershina E., Rudi K., Narbad A., Jenmalm M.C. (2015). The composition of the gut microbiota throughout life, with an emphasis on early life. Microb. Ecol. Health Dis..

[5] Madison A., Kiecolt-Glaser J.K. (2019). Stress, depression, diet, and the gut microbiota: Human-bacteria interactions at the core of psychoneuroimmunology and nutrition. Curr. Opin. Behav. Sci..

[6] Ronald Tyszkowski R.M. (2023). Chapter 3—Inflammation: A multifaceted and omnipresent phenomenon. Inflammation and Obesity.

[7] Sommer F., Anderson J.M., Bharti R., Raes J., Rosenstiel P. (2017). The resilience of the intestinal microbiota influences health and disease. Nat. Rev. Microbiol..

[8] Valdes A.M., Walter J., Segal E., Spector T.D. (2018). Role of the gut microbiota in nutrition and health. BMJ.

[9] Muller E., Algavi Y.M., Borenstein E. (2022). The gut microbiome-metabolome dataset collection: A curated resource for integrative meta-analysis. NPJ Biofilms Microbiomes.

[10] Zheng P., Zeng B., Zhou C., Liu M., Fang Z., Xu X., Zeng L., Chen J., Fan S., Du X. (2016). Gut microbiome remodeling induces depressive-like behaviors through a pathway mediated by the host’s metabolism. Mol. Psychiatry.

[11] Usami M., Kishimoto K., Ohata A., Miyoshi M., Aoyama M., Fueda Y., Kotani J. (2008). Butyrate and trichostatin A attenuate nuclear factor kappaB activation and tumor necrosis factor alpha secretion and increase prostaglandin E2 secretion in human peripheral blood mononuclear cells. Nutr. Res..

[12] Vinolo M.A., Rodrigues H.G., Hatanaka E., Sato F.T., Sampaio S.C., Curi R. (2011). Suppressive effect of short-chain fatty acids on production of proinflammatory mediators by neutrophils. J. Nutr. Biochem..

[13] Corthay A. (2009). How do regulatory T cells work?. Scand. J. Immunol..

[14] Lucas J.L., Mirshahpanah P., Haas-Stapleton E., Asadullah K., Zollner T.M., Numerof R.P. (2009). Induction of Foxp3+ regulatory T cells with histone deacetylase inhibitors. Cell Immunol..

[15] Erny D., Hrabe de Angelis A.L., Jaitin D., Wieghofer P., Staszewski O., David E., Keren-Shaul H., Mahlakoiv T., Jakobshagen K., Buch T. (2015). Host microbiota constantly control maturation and function of microglia in the CNS. Nat. Neurosci..

[16] Portincasa P., Bonfrate L., Vacca M., De Angelis M., Farella I., Lanza E., Khalil M., Wang D.Q., Sperandio M., Di Ciaula A. (2022). Gut Microbiota and Short Chain Fatty Acids: Implications in Glucose Homeostasis. Int. J. Mol. Sci..

[17] Gao K., Mu C.L., Farzi A., Zhu W.Y. (2020). Tryptophan Metabolism: A Link Between the Gut Microbiota and Brain. Adv. Nutr..

[18] Cervenka I., Agudelo L.Z., Ruas J.L. (2017). Kynurenines: Tryptophan’s metabolites in exercise, inflammation, and mental health. Science.

[19] Caspani G., Kennedy S., Foster J.A., Swann J. (2019). Gut microbial metabolites in depression: Understanding the biochemical mechanisms. Microb. Cell.

[20] Averina O.V., Zorkina Y.A., Yunes R.A., Kovtun A.S., Ushakova V.M., Morozova A.Y., Kostyuk G.P., Danilenko V.N., Chekhonin V.P. (2020). Bacterial Metabolites of Human Gut Microbiota Correlating with Depression. Int. J. Mol. Sci..

[21] Feehily C., Karatzas K.A. (2013). Role of glutamate metabolism in bacterial responses towards acid and other stresses. J. Appl. Microbiol..

[22] Pokusaeva K., Johnson C., Luk B., Uribe G., Fu Y., Oezguen N., Matsunami R.K., Lugo M., Major A., Mori-Akiyama Y. (2017). GABA-producing Bifidobacterium dentium modulates visceral sensitivity in the intestine. Neurogastroenterol. Motil..

[23] Sokovic Bajic S., Djokic J., Dinic M., Veljovic K., Golic N., Mihajlovic S., Tolinacki M. (2019). GABA-Producing Natural Dairy Isolate From Artisanal Zlatar Cheese Attenuates Gut Inflammation and Strengthens Gut Epithelial Barrier in vitro. Front. Microbiol..

[24] Hyland N.P., Cryan J.F. (2010). A Gut Feeling about GABA: Focus on GABA(B) Receptors. Front. Pharmacol..

[25] Hall V., Bendtsen K.M.S. (2023). Getting closer to modeling the gut-brain axis using induced pluripotent stem cells. Front. Cell Dev. Biol..

[26] Goodin D.S., Khankhanian P., Gourraud P.A., Vince N. (2021). Genetic susceptibility to multiple sclerosis: Interactions between conserved extended haplotypes of the MHC and other susceptibility regions. BMC Med. Genomics..

[27] Brocke S., Veromaa T., Weissman I.L., Gijbels K., Steinman L. (1994). Infection and multiple sclerosis: A possible role for superantigens?. Trends Microbiol..

[28] Filippi M., Bar-Or A., Piehl F., Preziosa P., Solari A., Vukusic S., Rocca M.A. (2018). Multiple sclerosis. Nat. Rev. Dis. Primers.

[29] Palumbo S., Pellegrini S., Zagon I.S., McLaughlin P.J. (2017). Experimental In Vivo Models of Multiple Sclerosis: State of the Art. Multiple Sclerosis: Perspectives in Treatment and Pathogenesis.

[30] Yokote H., Miyake S., Croxford J.L., Oki S., Mizusawa H., Yamamura T. (2008). NKT cell-dependent amelioration of a mouse model of multiple sclerosis by altering gut flora. Am. J. Pathol..

[31] Ochoa-Reparaz J., Mielcarz D.W., Ditrio L.E., Burroughs A.R., Foureau D.M., Haque-Begum S., Kasper L.H. (2009). Role of gut commensal microflora in the development of experimental autoimmune encephalomyelitis. J. Immunol..

[32] Seifert H.A., Benedek G., Nguyen H., Gerstner G., Zhang Y., Kent G., Vandenbark A.A., Bernhagen J., Offner H. (2018). Antibiotics protect against EAE by increasing regulatory and anti-inflammatory cells. Metab. Brain Dis..

[33] Stanisavljevic S., Cepic A., Bojic S., Veljovic K., Mihajlovic S., Dedovic N., Jevtic B., Momcilovic M., Lazarevic M., Mostarica Stojkovic M. (2019). Oral neonatal antibiotic treatment perturbs gut microbiota and aggravates central nervous system autoimmunity in Dark Agouti rats. Sci. Rep..

[34] Lee Y.K., Menezes J.S., Umesaki Y., Mazmanian S.K. (2011). Proinflammatory T-cell responses to gut microbiota promote experimental autoimmune encephalomyelitis. Proc. Natl. Acad. Sci. USA.

[35] Miyauchi E., Kim S.W., Suda W., Kawasumi M., Onawa S., Taguchi-Atarashi N., Morita H., Taylor T.D., Hattori M., Ohno H. (2020). Gut microorganisms act together to exacerbate inflammation in spinal cords. Nature.

[36] Radojevic D., Bekic M., Gruden-Movsesijan A., Ilic N., Dinic M., Bisenic A., Golic N., Vucevic D., Dokic J., Tomic S. (2022). Myeloid-derived suppressor cells prevent disruption of the gut barrier, preserve microbiota composition, and potentiate immunoregulatory pathways in a rat model of experimental autoimmune encephalomyelitis. Gut Microbes.

[37] Horton M.K., McCauley K., Fadrosh D., Fujimura K., Graves J., Ness J., Wheeler Y., Gorman M.P., Benson L.A., Weinstock-Guttman B. (2021). Gut microbiome is associated with multiple sclerosis activity in children. Ann. Clin. Transl. Neurol..

[38] Schepici G., Silvestro S., Bramanti P., Mazzon E. (2019). The Gut Microbiota in Multiple Sclerosis: An Overview of Clinical Trials. Cell Transplant..

[39] Chen J., Chia N., Kalari K.R., Yao J.Z., Novotna M., Paz Soldan M.M., Luckey D.H., Marietta E.V., Jeraldo P.R., Chen X. (2016). Multiple sclerosis patients have a distinct gut microbiota compared to healthy controls. Sci. Rep..

[40] Kujawa D., Laczmanski L., Budrewicz S., Pokryszko-Dragan A., Podbielska M. (2023). Targeting gut microbiota: New therapeutic opportunities in multiple sclerosis. Gut. Microbes..

[41] Duscha A., Gisevius B., Hirschberg S., Yissachar N., Stangl G.I., Dawin E., Bader V., Haase S., Kaisler J., David C. (2020). Propionic Acid Shapes the Multiple Sclerosis Disease Course by an Immunomodulatory Mechanism. Cell.

[42] Grant C.V., Loman B.R., Bailey M.T., Pyter L.M. (2021). Manipulations of the gut microbiome alter chemotherapy-induced inflammation and behavioral side effects in female mice. Brain Behav. Immun..

[43] Paytuvi-Gallart A., Sanseverino W., Winger A.M. (2020). Daily intake of probiotic strain Bacillus subtilis DE111 supports a healthy microbiome in children attending day-care. Benef. Microbes.

[44] Schirmer M., Smeekens S.P., Vlamakis H., Jaeger M., Oosting M., Franzosa E.A., Horst R.T., Jansen T., Jacobs L., Bonder M.J. (2016). Linking the Human Gut Microbiome to Inflammatory Cytokine Production Capacity. Cell.

[45] Cekanaviciute E., Yoo B.B., Runia T.F., Debelius J.W., Singh S., Nelson C.A., Kanner R., Bencosme Y., Lee Y.K., Hauser S.L. (2017). Gut bacteria from multiple sclerosis patients modulate human T cells and exacerbate symptoms in mouse models. Proc. Natl. Acad. Sci. USA.

[46] Breugelmans T., Oosterlinck B., Arras W., Ceuleers H., De Man J., Hold G.L., De Winter B.Y., Smet A. (2022). The role of mucins in gastrointestinal barrier function during health and disease. Lancet Gastroenterol. Hepatol..

[47] Wu W.K.K. (2023). Parabacteroides distasonis: An emerging probiotic?. Gut.

[48] Lehman P.C., Ghimire S., Price J.D., Ramer-Tait A.E., Mangalam A.K. (2023). Diet-microbiome-immune interplay in multiple sclerosis: Understanding the impact of phytoestrogen metabolizing gut bacteria. Eur. J. Immunol..

[49] Jensen S.N., Cady N.M., Shahi S.K., Peterson S.R., Gupta A., Gibson-Corley K.N., Mangalam A.K. (2021). Isoflavone diet ameliorates experimental autoimmune encephalomyelitis through modulation of gut bacteria depleted in patients with multiple sclerosis. Sci. Adv..

[50] Fransen F., van Beek A.A., Borghuis T., Meijer B., Hugenholtz F., van der Gaast-de Jongh C., Savelkoul H.F., de Jonge M.I., Faas M.M., Boekschoten M.V. (2017). The Impact of Gut Microbiota on Gender-Specific Differences in Immunity. Front. Immunol..

[51] Koren O., Goodrich J.K., Cullender T.C., Spor A., Laitinen K., Backhed H.K., Gonzalez A., Werner J.J., Angenent L.T., Knight R. (2012). Host remodeling of the gut microbiome and metabolic changes during pregnancy. Cell.

[52] Markle J.G., Frank D.N., Mortin-Toth S., Robertson C.E., Feazel L.M., Rolle-Kampczyk U., von Bergen M., McCoy K.D., Macpherson A.J., Danska J.S. (2013). Sex differences in the gut microbiome drive hormone-dependent regulation of autoimmunity. Science.

[53] Cox L.M., Abou-El-Hassan H., Maghzi A.H., Vincentini J., Weiner H.L. (2019). The sex-specific interaction of the microbiome in neurodegenerative diseases. Brain Res..

[54] Benedek G., Zhang J., Nguyen H., Kent G., Seifert H.A., Davin S., Stauffer P., Vandenbark A.A., Karstens L., Asquith M. (2017). Estrogen protection against EAE modulates the microbiota and mucosal-associated regulatory cells. J. Neuroimmunol..

[55] Spence R.D., Wisdom A.J., Cao Y., Hill H.M., Mongerson C.R., Stapornkul B., Itoh N., Sofroniew M.V., Voskuhl R.R. (2013). Estrogen mediates neuroprotection and anti-inflammatory effects during EAE through ERalpha signaling on astrocytes but not through ERbeta signaling on astrocytes or neurons. J. Neurosci..

[56] Bains N., Abdijadid S. (2024). Major Depressive Disorder. StatPearls.

[57] Reyes-Martinez S., Segura-Real L., Gomez-Garcia A.P., Tesoro-Cruz E., Constantino-Jonapa L.A., Amedei A., Aguirre-Garcia M.M. (2023). Neuroinflammation, Microbiota-Gut-Brain Axis, and Depression: The Vicious Circle. J. Integr. Neurosci..

[58] Remes O., Mendes J.F., Templeton P. (2021). Biological, Psychological, and Social Determinants of Depression: A Review of Recent Literature. Brain Sci..

[59] Rhie S.J., Jung E.Y., Shim I. (2020). The role of neuroinflammation on pathogenesis of affective disorders. J. Exerc. Rehabil..

[60] Chaudhry T.S., Senapati S.G., Gadam S., Mannam H., Voruganti H.V., Abbasi Z., Abhinav T., Challa A.B., Pallipamu N., Bheemisetty N. (2023). The Impact of Microbiota on the Gut-Brain Axis: Examining the Complex Interplay and Implications. J. Clin. Med..

[61] Williams B.B., Van Benschoten A.H., Cimermancic P., Donia M.S., Zimmermann M., Taketani M., Ishihara A., Kashyap P.C., Fraser J.S., Fischbach M.A. (2014). Discovery and characterization of gut microbiota decarboxylases that can produce the neurotransmitter tryptamine. Cell Host Microbe.

[62] Zheng P., Yang J., Li Y., Wu J., Liang W., Yin B., Tan X., Huang Y., Chai T., Zhang H. (2020). Gut Microbial Signatures Can Discriminate Unipolar from Bipolar Depression. Adv. Sci..

[63] Liu L., Wang H., Zhang H., Chen X., Zhang Y., Wu J., Zhao L., Wang D., Pu J., Ji P. (2022). Toward a Deeper Understanding of Gut Microbiome in Depression: The Promise of Clinical Applicability. Adv. Sci..

[64] Nikolova V.L., Smith M.R.B., Hall L.J., Cleare A.J., Stone J.M., Young A.H. (2021). Perturbations in Gut Microbiota Composition in Psychiatric Disorders: A Review and Meta-analysis. JAMA Psychiatry.

[65] Simpson C.A., Diaz-Arteche C., Eliby D., Schwartz O.S., Simmons J.G., Cowan C.S.M. (2021). The gut microbiota in anxiety and depression—A systematic review. Clin. Psychol. Rev..

[66] Chen Z., Li J., Gui S., Zhou C., Chen J., Yang C., Hu Z., Wang H., Zhong X., Zeng L. (2018). Comparative metaproteomics analysis shows altered fecal microbiota signatures in patients with major depressive disorder. Neuroreport.

[67] Jiang H., Ling Z., Zhang Y., Mao H., Ma Z., Yin Y., Wang W., Tang W., Tan Z., Shi J. (2015). Altered fecal microbiota composition in patients with major depressive disorder. Brain Behav. Immun..

[68] Kelly J.R., Borre Y., O’Brien C., Patterson E., El Aidy S., Deane J., Kennedy P.J., Beers S., Scott K., Moloney G. (2016). Transferring the blues: Depression-associated gut microbiota induces neurobehavioural changes in the rat. J. Psychiatr. Res..

[69] Chung Y.E., Chen H.C., Chou H.L., Chen I.M., Lee M.S., Chuang L.C., Liu Y.W., Lu M.L., Chen C.H., Wu C.S. (2019). Exploration of microbiota targets for major depressive disorder and mood related traits. J. Psychiatr. Res..

[70] Lai W.T., Deng W.F., Xu S.X., Zhao J., Xu D., Liu Y.H., Guo Y.Y., Wang M.B., He F.S., Ye S.W. (2021). Shotgun metagenomics reveals both taxonomic and tryptophan pathway differences of gut microbiota in major depressive disorder patients. Psychol. Med..

[71] Rong H., Xie X.H., Zhao J., Lai W.T., Wang M.B., Xu D., Liu Y.H., Guo Y.Y., Xu S.X., Deng W.F. (2019). Similarly in depression, nuances of gut microbiota: Evidences from a shotgun metagenomics sequencing study on major depressive disorder versus bipolar disorder with current major depressive episode patients. J. Psychiatr. Res..

[72] Huang Y., Shi X., Li Z., Shen Y., Shi X., Wang L., Li G., Yuan Y., Wang J., Zhang Y. (2018). Possible association of Firmicutes in the gut microbiota of patients with major depressive disorder. Neuropsychiatr. Dis. Treat..

[73] Liu Y., Zhang L., Wang X., Wang Z., Zhang J., Jiang R., Wang X., Wang K., Liu Z., Xia Z. (2016). Similar Fecal Microbiota Signatures in Patients With Diarrhea-Predominant Irritable Bowel Syndrome and Patients With Depression. Clin. Gastroenterol. Hepatol..

[74] Valles-Colomer M., Falony G., Darzi Y., Tigchelaar E.F., Wang J., Tito R.Y., Schiweck C., Kurilshikov A., Joossens M., Wijmenga C. (2019). The neuroactive potential of the human gut microbiota in quality of life and depression. Nat. Microbiol..

[75] Clarke G., Grenham S., Scully P., Fitzgerald P., Moloney R.D., Shanahan F., Dinan T.G., Cryan J.F. (2013). The microbiome-gut-brain axis during early life regulates the hippocampal serotonergic system in a sex-dependent manner. Mol. Psychiatry.

[76] Liu L., Wang H., Chen X., Zhang Y., Zhang H., Xie P. (2023). Gut microbiota and its metabolites in depression: From pathogenesis to treatment. eBioMedicine.

[77] Frost G., Sleeth M.L., Sahuri-Arisoylu M., Lizarbe B., Cerdan S., Brody L., Anastasovska J., Ghourab S., Hankir M., Zhang S. (2014). The short-chain fatty acid acetate reduces appetite via a central homeostatic mechanism. Nat. Commun..

[78] Kim S.J., Lee H., Lee G., Oh S.J., Shin M.K., Shim I., Bae H. (2012). CD4+CD25+ regulatory T cell depletion modulates anxiety and depression-like behaviors in mice. PLoS ONE.

[79] Sun N., Zhang J., Wang J., Liu Z., Wang X., Kang P., Yang C., Liu P., Zhang K. (2022). Abnormal gut microbiota and bile acids in patients with first-episode major depressive disorder and correlation analysis. Psychiatry Clin. Neurosci..

[80] Meinitzer S., Baranyi A., Holasek S., Schnedl W.J., Zelzer S., Mangge H., Herrmann M., Meinitzer A., Enko D. (2020). Sex-Specific Associations of Trimethylamine-N-Oxide and Zonulin with Signs of Depression in Carbohydrate Malabsorbers and Nonmalabsorbers. Dis. Markers.

[81] Liu Y.W., Liu W.H., Wu C.C., Juan Y.C., Wu Y.C., Tsai H.P., Wang S., Tsai Y.C. (2016). Psychotropic effects of Lactobacillus plantarum PS128 in early life-stressed and naive adult mice. Brain Res..

[82] Gilbert K., Arseneault-Breard J., Flores Monaco F., Beaudoin A., Bah T.M., Tompkins T.A., Godbout R., Rousseau G. (2013). Attenuation of post-myocardial infarction depression in rats by n-3 fatty acids or probiotics starting after the onset of reperfusion. Br. J. Nutr..

[83] Callaghan B.L., Cowan C.S., Richardson R. (2016). Treating Generational Stress: Effect of Paternal Stress on Development of Memory and Extinction in Offspring Is Reversed by Probiotic Treatment. Psychol. Sci..

[84] Hao Z., Wang W., Guo R., Liu H. (2019). Faecalibacterium prausnitzii (ATCC 27766) has preventive and therapeutic effects on chronic unpredictable mild stress-induced depression-like and anxiety-like behavior in rats. Psychoneuroendocrinology.

[85] Tian P., Chen Y., Zhu H., Wang L., Qian X., Zou R., Zhao J., Zhang H., Qian L., Wang Q. (2022). Bifidobacterium breve CCFM1025 attenuates major depression disorder via regulating gut microbiome and tryptophan metabolism: A randomized clinical trial. Brain Behav. Immun..

[86] Zhang X., Chen S., Zhang M., Ren F., Ren Y., Li Y., Liu N., Zhang Y., Zhang Q., Wang R. (2021). Effects of Fermented Milk Containing Lacticaseibacillus paracasei Strain Shirota on Constipation in Patients with Depression: A Randomized, Double-Blind, Placebo-Controlled Trial. Nutrients.

[87] Slykerman R.F., Hood F., Wickens K., Thompson J.M.D., Barthow C., Murphy R., Kang J., Rowden J., Stone P., Crane J. (2017). Effect of Lactobacillus rhamnosus HN001 in Pregnancy on Postpartum Symptoms of Depression and Anxiety: A Randomised Double-blind Placebo-controlled Trial. eBioMedicine.

[88] Schaub A.C., Schneider E., Vazquez-Castellanos J.F., Schweinfurth N., Kettelhack C., Doll J.P.K., Yamanbaeva G., Mahlmann L., Brand S., Beglinger C. (2022). Clinical, gut microbial and neural effects of a probiotic add-on therapy in depressed patients: A randomized controlled trial. Transl. Psychiatry.

[89] Miyaoka T., Kanayama M., Wake R., Hashioka S., Hayashida M., Nagahama M., Okazaki S., Yamashita S., Miura S., Miki H. (2018). Clostridium butyricum MIYAIRI 588 as Adjunctive Therapy for Treatment-Resistant Major Depressive Disorder: A Prospective Open-Label Trial. Clin. Neuropharmacol..

[90] Messaoudi M., Lalonde R., Violle N., Javelot H., Desor D., Nejdi A., Bisson J.F., Rougeot C., Pichelin M., Cazaubiel M. (2011). Assessment of psychotropic-like properties of a probiotic formulation (Lactobacillus helveticus R0052 and Bifidobacterium longum R0175) in rats and human subjects. Br. J. Nutr..

[91] Kazemi A., Noorbala A.A., Azam K., Eskandari M.H., Djafarian K. (2019). Effect of probiotic and prebiotic vs placebo on psychological outcomes in patients with major depressive disorder: A randomized clinical trial. Clin. Nutr..

[92] Akkasheh G., Kashani-Poor Z., Tajabadi-Ebrahimi M., Jafari P., Akbari H., Taghizadeh M., Memarzadeh M.R., Asemi Z., Esmaillzadeh A. (2016). Clinical and metabolic response to probiotic administration in patients with major depressive disorder: A randomized, double-blind, placebo-controlled trial. Nutrition.

[93] Musazadeh V., Zarezadeh M., Faghfouri A.H., Keramati M., Jamilian P., Jamilian P., Mohagheghi A., Farnam A. (2023). Probiotics as an effective therapeutic approach in alleviating depression symptoms: An umbrella meta-analysis. Crit. Rev. Food Sci. Nutr..

[94] Guo T., Zhang D., Zeng Y., Huang T.Y., Xu H., Zhao Y. (2020). Molecular and cellular mechanisms underlying the pathogenesis of Alzheimer’s disease. Mol. Neurodegener..

[95] Nardini E., Hogan R., Flamier A., Bernier G. (2021). Alzheimer’s disease: A tale of two diseases?. Neural Regen. Res..

[96] Raulin A.C., Doss S.V., Trottier Z.A., Ikezu T.C., Bu G., Liu C.C. (2022). ApoE in Alzheimer’s disease: Pathophysiology and therapeutic strategies. Mol. Neurodegener..

[97] Ghosh T.S., Shanahan F., O’Toole P.W. (2022). The gut microbiome as a modulator of healthy ageing. Nat. Rev. Gastroenterol. Hepatol..

[98] Sahlgren Bendtsen K.M., Hall V.J. (2023). The Breakthroughs and Caveats of Using Human Pluripotent Stem Cells in Modeling Alzheimer’s Disease. Cells.

[99] Chandra S., Sisodia S.S., Vassar R.J. (2023). The gut microbiome in Alzheimer’s disease: What we know and what remains to be explored. Mol. Neurodegener..

[100] Ferreiro A.L., Choi J., Ryou J., Newcomer E.P., Thompson R., Bollinger R.M., Hall-Moore C., Ndao I.M., Sax L., Benzinger T.L.S. (2023). Gut microbiome composition may be an indicator of preclinical Alzheimer’s disease. Sci. Transl. Med..

[101] Zhuang Z.Q., Shen L.L., Li W.W., Fu X., Zeng F., Gui L., Lu Y., Cai M., Zhu C., Tan Y.L. (2018). Gut Microbiota is Altered in Patients with Alzheimer’s Disease. J. Alzheimer’s Dis..

[102] Vogt N.M., Kerby R.L., Dill-McFarland K.A., Harding S.J., Merluzzi A.P., Johnson S.C., Carlsson C.M., Asthana S., Zetterberg H., Blennow K. (2017). Gut microbiome alterations in Alzheimer’s disease. Sci. Rep..

[103] Gao C., Li B., He Y., Huang P., Du J., He G., Zhang P., Tang H., Chen S. (2023). Early changes of fecal short-chain fatty acid levels in patients with mild cognitive impairments. CNS Neurosci. Ther..

[104] Chen C., Ahn E.H., Kang S.S., Liu X., Alam A., Ye K. (2020). Gut dysbiosis contributes to amyloid pathology, associated with C/EBPbeta/AEP signaling activation in Alzheimer’s disease mouse model. Sci. Adv..

[105] Zhang L., Wang Y., Xiayu X., Shi C., Chen W., Song N., Fu X., Zhou R., Xu Y.F., Huang L. (2017). Altered Gut Microbiota in a Mouse Model of Alzheimer’s Disease. J. Alzheimer’s Dis..

[106] Kim N., Jeon S.H., Ju I.G., Gee M.S., Do J., Oh M.S., Lee J.K. (2021). Transplantation of gut microbiota derived from Alzheimer’s disease mouse model impairs memory function and neurogenesis in C57BL/6 mice. Brain Behav. Immun..

[107] Seo D.O., O’Donnell D., Jain N., Ulrich J.D., Herz J., Li Y., Lemieux M., Cheng J., Hu H., Serrano J.R. (2023). ApoE isoform- and microbiota-dependent progression of neurodegeneration in a mouse model of tauopathy. Science.

[108] Dalile B., Van Oudenhove L., Vervliet B., Verbeke K. (2019). The role of short-chain fatty acids in microbiota-gut-brain communication. Nat. Rev. Gastroenterol. Hepatol..

[109] MahmoudianDehkordi S., Arnold M., Nho K., Ahmad S., Jia W., Xie G., Louie G., Kueider-Paisley A., Moseley M.A., Thompson J.W. (2019). Altered bile acid profile associates with cognitive impairment in Alzheimer’s disease-An emerging role for gut microbiome. Alzheimer’s Dement..

[110] Bendheim P.E., Poeggeler B., Neria E., Ziv V., Pappolla M.A., Chain D.G. (2002). Development of indole-3-propionic acid (OXIGON) for Alzheimer’s disease. J. Mol. Neurosci..

[111] Abdelhamid M., Zhou C., Ohno K., Kuhara T., Taslima F., Abdullah M., Jung C.G., Michikawa M. (2022). Probiotic Bifidobacterium breve Prevents Memory Impairment Through the Reduction of Both Amyloid-beta Production and Microglia Activation in APP Knock-In Mouse. J. Alzheimer’s Dis..

[112] Cogliati S., Clementi V., Francisco M., Crespo C., Arganaraz F., Grau R. (2020). Bacillus Subtilis Delays Neurodegeneration and Behavioral Impairment in the Alzheimer’s Disease Model Caenorhabditis Elegans. J. Alzheimer’s Dis..

[113] Hughes H.K., Moreno R.J., Ashwood P. (2024). Innate Immune Dysfunction and Neuroinflammation in Autism Spectrum Disorder (ASD). Focus.

[114] Hirota T., King B.H. (2023). Autism Spectrum Disorder: A Review. JAMA.

[115] Holingue C., Newill C., Lee L.C., Pasricha P.J., Daniele Fallin M. (2018). Gastrointestinal symptoms in autism spectrum disorder: A review of the literature on ascertainment and prevalence. Autism. Res..

[116] Leader G., Abberton C., Cunningham S., Gilmartin K., Grudzien M., Higgins E., Joshi L., Whelan S., Mannion A. (2022). Gastrointestinal Symptoms in Autism Spectrum Disorder: A Systematic Review. Nutrients.

[117] Iglesias-Vazquez L., Van Ginkel Riba G., Arija V., Canals J. (2020). Composition of Gut Microbiota in Children with Autism Spectrum Disorder: A Systematic Review and Meta-Analysis. Nutrients.

[118] Yang C., Xiao H., Zhu H., Du Y., Wang L. (2024). Revealing the gut microbiome mystery: A meta-analysis revealing differences between individuals with autism spectrum disorder and neurotypical children. Biosci. Trends.

[119] Morton J.T., Jin D.M., Mills R.H., Shao Y., Rahman G., McDonald D., Zhu Q., Balaban M., Jiang Y., Cantrell K. (2023). Multi-level analysis of the gut-brain axis shows autism spectrum disorder-associated molecular and microbial profiles. Nat. Neurosci..

[120] Peralta-Marzal L.N., Rojas-Velazquez D., Rigters D., Prince N., Garssen J., Kraneveld A.D., Perez-Pardo P., Lopez-Rincon A. (2024). A robust microbiome signature for autism spectrum disorder across different studies using machine learning. Sci. Rep..

[121] Kang D.W., Adams J.B., Coleman D.M., Pollard E.L., Maldonado J., McDonough-Means S., Caporaso J.G., Krajmalnik-Brown R. (2019). Long-term benefit of Microbiota Transfer Therapy on autism symptoms and gut microbiota. Sci. Rep..

[122] Kang D.W., Adams J.B., Gregory A.C., Borody T., Chittick L., Fasano A., Khoruts A., Geis E., Maldonado J., McDonough-Means S. (2017). Microbiota Transfer Therapy alters gut ecosystem and improves gastrointestinal and autism symptoms: An open-label study. Microbiome.

[123] Li N., Chen H., Cheng Y., Xu F., Ruan G., Ying S., Tang W., Chen L., Chen M., Lv L. (2021). Fecal Microbiota Transplantation Relieves Gastrointestinal and Autism Symptoms by Improving the Gut Microbiota in an Open-Label Study. Front. Cell Infect. Microbiol..

[124] Brzoska-Konkol E., Remberk B., Papasz-Siemienuk A. (2022). Analysis of research on the effectiveness of using probiotics for children with autism spectrum disorders, in order to reduce the core and accompanying autism symptoms. Review of randomized clinical trials. Postep. Psychiatr. Neurol..

[125] Santocchi E., Guiducci L., Prosperi M., Calderoni S., Gaggini M., Apicella F., Tancredi R., Billeci L., Mastromarino P., Grossi E. (2020). Effects of Probiotic Supplementation on Gastrointestinal, Sensory and Core Symptoms in Autism Spectrum Disorders: A Randomized Controlled Trial. Front. Psychiatry.

[126] Billeci L., Callara A.L., Guiducci L., Prosperi M., Morales M.A., Calderoni S., Muratori F., Santocchi E. (2023). A randomized controlled trial into the effects of probiotics on electroencephalography in preschoolers with autism. Autism.

[127] Hill C., Guarner F., Reid G., Gibson G.R., Merenstein D.J., Pot B., Morelli L., Canani R.B., Flint H.J., Salminen S. (2014). Expert consensus document. The International Scientific Association for Probiotics and Prebiotics consensus statement on the scope and appropriate use of the term probiotic. Nat. Rev. Gastroenterol. Hepatol..

[128] Rajilić-Stojanović M. (2019). , Dimitrijević S., Golić, N. Lactic Acid Bacteria in the Gut.

[129] Terzic-Vidojevic A., Veljovic K., Tolinacki M., Zivkovic M., Lukic J., Lozo J., Fira D., Jovcic B., Strahinic I., Begovic J. (2020). Diversity of non-starter lactic acid bacteria in autochthonous dairy products from Western Balkan Countries—Technological and probiotic properties. Food Res. Int..

[130] Vinderola G., Ouwehand A., Salminen S., Wright A. (2019). Lactic Acid Bacteria Microbiological and Functional Aspects.

[131] Dinic M., Jakovljevic S., Dokic J., Popovic N., Radojevic D., Strahinic I., Golic N. (2021). Probiotic-mediated p38 MAPK immune signaling prolongs the survival of Caenorhabditis elegans exposed to pathogenic bacteria. Sci. Rep..

[132] Stankovic M., Veljovic K., Popovic N., Kojic S., Dunjic Manevski S., Radojkovic D., Golic N. (2022). Lactobacillus brevis BGZLS10-17 and Lb. plantarum BGPKM22 Exhibit Anti-Inflammatory Effect by Attenuation of NF-kappaB and MAPK Signaling in Human Bronchial Epithelial Cells. Int. J. Mol. Sci..

[133] Terzic-Vidojevic A., Veljovic K., Popovic N., Tolinacki M., Golic N. (2021). Enterococci from Raw-Milk Cheeses: Current Knowledge on Safety, Technological, and Probiotic Concerns. Foods.

[134] Babic M., Veljovic K., Popovic N., Golic N., Radojkovic D., Stankovic M. (2023). Antioxidant effect of lactic acid bacteria in human bronchial epithelial cells exposed to cigarette smoke. J. Appl. Microbiol..

[135] Brdaric E., Popovic D., Sokovic Bajic S., Tucovic D., Mutic J., Cakic-Milosevic M., Durdic S., Tolinacki M., Aleksandrov A.P., Golic N. (2023). Orally Administrated Lactiplantibacillus plantarum BGAN8-Derived EPS-AN8 Ameliorates Cd Hazards in Rats. Int. J. Mol. Sci..

[136] Brdaric E., Sokovic Bajic S., Dokic J., Durdic S., Ruas-Madiedo P., Stevanovic M., Tolinacki M., Dinic M., Mutic J., Golic N. (2021). Protective Effect of an Exopolysaccharide Produced by Lactiplantibacillus plantarum BGAN8 Against Cadmium-Induced Toxicity in Caco-2 Cells. Front. Microbiol..

[137] Mihailovic M., Sokovic Bajic S., Arambasic Jovanovic J., Brdaric E., Dinic S., Grdovic N., Uskokovic A., Rajic J., Dordevic M., Tolinacki M. (2024). Beneficial Effects of Probiotic Lactobacillus paraplantarum BGCG11 on Pancreatic and Duodenum Function in Diabetic Rats. Int. J. Mol. Sci..

[138] Mihailović M., Živković M., Jovanović J.A., Tolinački M., Sinadinović M., Rajić J., Uskoković A., Dinić S., Grdović N., Golić N. (2017). Oral administration of probiotic Lactobacillus paraplantarum BGCG11 attenuates diabetes-induced liver and kidney damage in rats. J. Funct. Foods.

[139] Dinic M., Herholz M., Kacarevic U., Radojevic D., Novovic K., Dokic J., Trifunovic A., Golic N. (2021). Host-commensal interaction promotes health and lifespan in Caenorhabditis elegans through the activation of HLH-30/TFEB-mediated autophagy. Aging.

[140] Dinic M., Jakovljevic S., Popovic N., Radojevic D., Veljovic K., Golic N., Terzic-Vidojevic A. (2023). Assessment of stability and bioactive compounds in yogurt containing novel natural starter cultures with the ability to promote longevity in Caenorhabditis elegans. J. Dairy Sci..

[141] Lagier J.C., Dubourg G., Million M., Cadoret F., Bilen M., Fenollar F., Levasseur A., Rolain J.M., Fournier P.E., Raoult D. (2018). Culturing the human microbiota and culturomics. Nat. Rev. Microbiol..

[142] Wan X., Yang Q., Wang X., Bai Y., Liu Z. (2023). Isolation and Cultivation of Human Gut Microorganisms: A Review. Microorganisms.

[143] Azkona G., Sanchez-Pernaute R. (2022). Mice in translational neuroscience: What R we doing?. Prog. Neurobiol..

[144] Wu D., Chen Q., Chen X., Han F., Chen Z., Wang Y. (2023). The blood-brain barrier: Structure, regulation, and drug delivery. Signal. Transduct. Target. Ther..

[145] Stone N.L., England T.J., O’Sullivan S.E. (2019). A Novel Transwell Blood Brain Barrier Model Using Primary Human Cells. Front. Cell Neurosci..

[146] Lopez-Tobon A., Shyti R., Villa C.E., Cheroni C., Fuentes-Bravo P., Trattaro S., Caporale N., Troglio F., Tenderini E., Mihailovich M. (2023). GTF2I dosage regulates neuronal differentiation and social behavior in 7q11.23 neurodevelopmental disorders. Sci. Adv..

[147] Mihailovich M., Germain P.L., Shyti R., Pozzi D., Noberini R., Liu Y., Aprile D., Tenderini E., Troglio F., Trattaro S. (2024). Multiscale modeling uncovers 7q11.23 copy number variation-dependent changes in ribosomal biogenesis and neuronal maturation and excitability. J. Clin. Investig..

[148] Danku A.E., Dulf E.H., Braicu C., Jurj A., Berindan-Neagoe I. (2022). Organ-On-A-Chip: A Survey of Technical Results and Problems. Front. Bioeng. Biotechnol..

[149] Hubatsch I., Ragnarsson E.G., Artursson P. (2007). Determination of drug permeability and prediction of drug absorption in Caco-2 monolayers. Nat. Protoc..

[150] Tan H.Y., Trier S., Rahbek U.L., Dufva M., Kutter J.P., Andresen T.L. (2018). A multi-chamber microfluidic intestinal barrier model using Caco-2 cells for drug transport studies. PLoS ONE.

[151] Kim H.J., Huh D., Hamilton G., Ingber D.E. (2012). Human gut-on-a-chip inhabited by microbial flora that experiences intestinal peristalsis-like motions and flow. Lab A Chip.

[152] Wang L., Han J., Su W., Li A., Zhang W., Li H., Hu H., Song W., Xu C., Chen J. (2023). Gut-on-a-chip for exploring the transport mechanism of Hg(II). Microsyst. Nanoeng..

[153] Jalili-Firoozinezhad S., Gazzaniga F.S., Calamari E.L., Camacho D.M., Fadel C.W., Bein A., Swenor B., Nestor B., Cronce M.J., Tovaglieri A. (2019). A complex human gut microbiome cultured in an anaerobic intestine-on-a-chip. Nat. Biomed. Eng..

[154] Workman M.J., Svendsen C.N. (2020). Recent advances in human iPSC-derived models of the blood-brain barrier. Fluids Barriers CNS.

[155] Vatine G.D., Barrile R., Workman M.J., Sances S., Barriga B.K., Rahnama M., Barthakur S., Kasendra M., Lucchesi C., Kerns J. (2019). Human iPSC-Derived Blood-Brain Barrier Chips Enable Disease Modeling and Personalized Medicine Applications. Cell Stem Cell.

[156] Pediaditakis I., Kodella K.R., Manatakis D.V., Le C.Y., Hinojosa C.D., Tien-Street W., Manolakos E.S., Vekrellis K., Hamilton G.A., Ewart L. (2021). Modeling alpha-synuclein pathology in a human brain-chip to assess blood-brain barrier disruption. Nat. Commun..

[157] Kim M.-H., Kim D., Sung J.H. (2021). A Gut-Brain Axis-on-a-Chip for studying transport across epithelial and endothelial barriers. J. Ind. Eng. Chem..

[158] Shin Y., Choi S.H., Kim E., Bylykbashi E., Kim J.A., Chung S., Kim D.Y., Kamm R.D., Tanzi R.E. (2019). Blood-Brain Barrier Dysfunction in a 3D In Vitro Model of Alzheimer’s Disease. Adv. Sci..

[159] Ng B., Rowland H.A., Wei T., Arunasalam K., Hayes E.M., Koychev I., Hedegaard A., Ribe E.M., Chan D., Chessell T. (2022). Neurons derived from individual early Alzheimer’s disease patients reflect their clinical vulnerability. Brain Commun..

[160] Faal T., Phan D.T.T., Davtyan H., Scarfone V.M., Varady E., Blurton-Jones M., Hughes C.C.W., Inlay M.A. (2019). Induction of Mesoderm and Neural Crest-Derived Pericytes from Human Pluripotent Stem Cells to Study Blood-Brain Barrier Interactions. Stem Cell Rep..

[161] Lu T.M., Houghton S., Magdeldin T., Duran J.G.B., Minotti A.P., Snead A., Sproul A., Nguyen D.T., Xiang J., Fine H.A. (2021). Pluripotent stem cell-derived epithelium misidentified as brain microvascular endothelium requires ETS factors to acquire vascular fate. Proc. Natl. Acad. Sci. USA.

[162] Stebbins M.J., Gastfriend B.D., Canfield S.G., Lee M.S., Richards D., Faubion M.G., Li W.J., Daneman R., Palecek S.P., Shusta E.V. (2019). Human pluripotent stem cell-derived brain pericyte-like cells induce blood-brain barrier properties. Sci. Adv..

[163] Gisevius B., Duscha A., Poschmann G., Stuhler K., Motte J., Fisse A.L., Augustyniak S., Rehm A., Renk P., Bose C. (2024). Propionic acid promotes neurite recovery in damaged multiple sclerosis neurons. Brain Commun..

